# Effect of the ten‐year fishing ban on change of phytoplankton community structure: Insights from the Gan River

**DOI:** 10.1002/ece3.70217

**Published:** 2024-08-29

**Authors:** Peng Ye, Xinwen Lu, Wenxin Xia, Yating Wang, Chunhua Zhou, Xiongjun Liu, Shan Ouyang, Xiaoping Wu

**Affiliations:** ^1^ School of Life Sciences Nanchang University Nanchang China; ^2^ Guangdong Provincial Key Laboratory of Conservation and Precision Utilization of Characteristic Agricultural Resources in Mountainous Areas, School of Life Sciences Jiaying University Meizhou China

**Keywords:** biodiversity, community structure, environmental change, functional group traits, phytoplankton

## Abstract

The Yangtze River is one of the largest riverine ecosystems in the world and is a biodiversity hotspot. In recent years, this river ecosystem has undergone rapid habitat deterioration due to anthropogenic disturbances, leading to a decrease in freshwater biodiversity. Owing to these anthropogenic impacts, the Chinese government imposed a “Ten‐year fishing ban” (TYFB) in the Yangtze River and its tributaries. Ecological changes associated with this decision have not been documented to evaluate the level of success. This study evaluates the success of the TYFB by analyzing the changes in phytoplankton communities and comparing them to periods before the TYFB was imposed. A total of 325 phytoplankton species belonging to 7 phyla and 103 genera dominated by Chlorophyceae and Bacillariophyceae were identified. Species diversity according to the Shannon–Wiener ranged between 1.19 and 3.00. The results indicated that phytoplankton diversity increased, while both density and biomass decreased after the TYFB. The health of the aquatic ecosystem appeared to have improved after the TYFB, as indicated by the phytoplankton‐based index of biotic integrity. Significant seasonal and spatial differences were found among the number of species, density, and biomass of phytoplankton, where these differences were correlated with pH, water depth, chlorophyll‐a, permanganate index, transparency, copper, ammonia nitrogen, and total phosphorus based on redundancy analysis. Results from this study indicate that the TYFB played an important role in the restoration of freshwater ecosystem in the Yangtze River and its tributaries.

## Graphical abstract

The present study disentangled the mechanisms driving the seasonal and spatial patterns in the phytoplankton community structure, showing the response of environmental factors after the TYFB. Seasonal changes, spatial patterns, and environmental changes were the main driving factors of the phytoplankton community structure. The health status of aquatic ecosystem showed a tendency to improve after the TYFB. These results indicate that the TYFB plays an important role in the restoration of freshwater ecosystem, and also more attention need to be focused on anthropogenic habitat alteration.

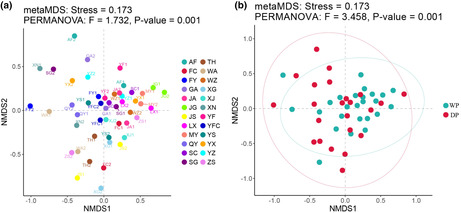

## 1 INTRODUCTION

Freshwater ecosystems play a critical role in social development (Bowes et al., 2018; Carpenter et al., 2011). There is only <1% of the planet surface in freshwater ecosystems, but they are invaluable for biodiversity with about 10% of all known species (Altermatt et al., 2013; Strayer & Dudgeon, 2010). However, anthropogenic disturbances, such as dam construction, habitat fragmentation, pollution, and exotic species invasions, have affected freshwater ecosystems, resulting in a decline in global biodiversity and loss of ecosystem function (Azan et al., 2015; Dudgeon, 2019; Reid et al., 2019; Waldron et al., 2017). To protect and restore freshwater biodiversity and ecosystem function, there is a need to analyze the change and driving mechanisms of aquatic community structure under intense anthropogenic disturbance (Dudgeon, 2019; Reid et al., 2019).

The Yangtze River is one of the largest riverine systems in the world and is a freshwater biodiversity hotspot (Liu et al., 2019; Liu & Wang, 2018). In recent years, this river ecosystem has undergone rapid habitat deterioration due to anthropogenic disturbances, leading to a decrease in freshwater biodiversity (Lu et al., 2021; Xie, 2017). For example, overfishing led to the decline in fish biodiversity (Liu et al., 2019, 2023; Mota et al., 2014), which affected the trophic relationships of food webs and the ecosystem stability (Mak et al., 2021; Wang et al., 2022). Closure of fishing has been proposed as a strategy to mitigate the effects of overfishing and to promote the recovery of fish stocks in many countries (Carvalho et al., 2019; Liu et al., 2023; Mak et al., 2021; Tuset et al., 2021; Wang et al., 2022; Xie et al., 2022). To improve the integrity of the freshwater ecosystem, China enacted the Yangtze River Protection Law in 2021 and imposed the “Ten‐year fishing ban” (TYFB). However, the effect of the TYFB on change of aquatic community structure remains unclear.

Phytoplankton, as the foundation of the pelagic food web and primary producers in freshwater ecosystems, are highly sensitive to environmental changes. This sensitivity reflects the dynamics and nutrient status of the ecosystem (Carvalho et al., 2013; Wang et al., 2021) and plays a crucial role in maintaining the stability and functioning of freshwater environments (Padisák et al., 2006). Maintaining the mechanism of phytoplankton community structure has been a key issue in freshwater ecosystems (Chaparro et al., 2018; Guelzow et al., 2016; Huszar et al., 2015). Changes of phytoplankton community structure and functional groups significantly affect the function of freshwater ecosystems (Carvalho et al., 2013; Pasztaleniec, 2016). For example, change of phytoplankton diversity and assemblages reflect a changing fish population in river ecosystems, and what that means for river conservation and restoration (Wang et al., 2022). Therefore, knowledge of the change and driving mechanisms of phytoplankton community structure are critical for protecting biodiversity and ecosystem function in freshwater ecosystems (Carvalho et al., 2013; Pasztaleniec, 2016).

The Gan River is one of the major tributaries of the Yangtze River and also plays a critical role in maintaining freshwater biodiversity for the Yangtze River (Guo et al., 2018; Li et al., 2019; Xie, 2017). There is no doubt that the Gan River faces the same environmental problems as the Yangtze River. Although previous studies have analyzed the phytoplankton community structure in the Gan River before the TYFB (Chen et al., 2009; Feng et al., 2021; Hu & Lin, 1988; Ji et al., 2012; Liu et al., 2012; Yang et al., 2020; Zhang et al., 2011), the change and driving mechanisms of phytoplankton community structure after the TYFB have not been documented. Here, we aim to: (1) analyze the seasonal and spatial changes of phytoplankton community structure in the middle and lower reaches of the Gan River Basin after the TYFB; (2) analyze the correlation between the phytoplankton community structure and the environmental factors; and (3) assess the ecosystem health based on the phytoplankton‐based index of biotic integrity (P‐IBI). We test whether the number of species, density, biomass, and community structure of phytoplankton have changed in response to TYFB and whether the patterns are predictable as a protection and restoration measure. This study provides an important reference for the management of ecosystem health and biodiversity conservation in freshwater ecosystem after the TYFB.

## 2 MATERIALS AND METHODS

### 2.1 Study area

**FIGURE 1:**
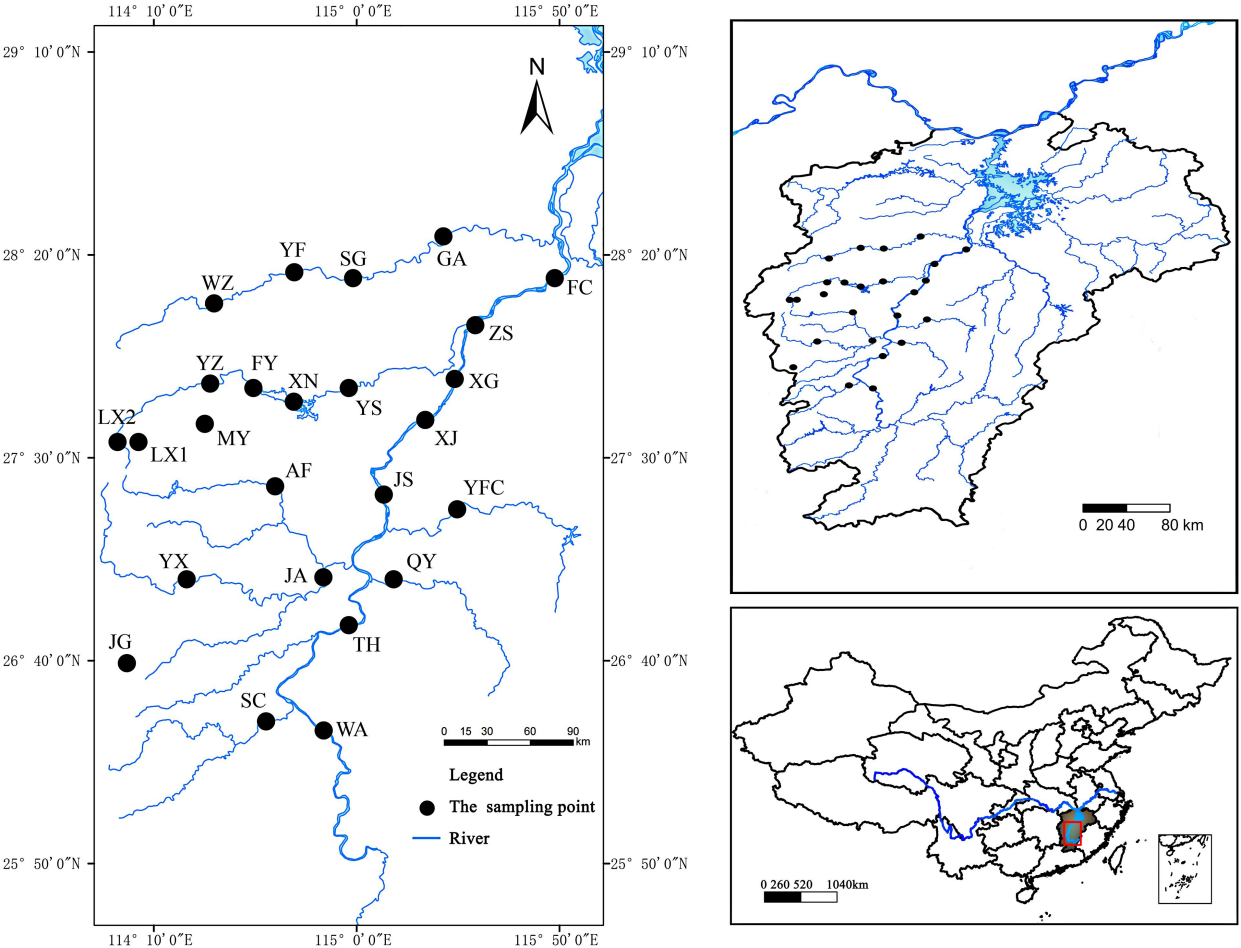
Map of the study area and sampling sites in the middle and lower reaches of the Gan River. Sampling site codes are as in Table S1.

The Gan River is one of the eight major tributaries of the Yangtze River and the largest river in Jiangxi Province, running through the whole Jiangxi Province from south to north, with a total length of 766 km in the main stream and a watershed area of 83,500 km^2^, and it is the main inlet of Poyang Lake (the largest freshwater lake in China). In recent decades, increased human activities have led to growing concerns about a range of ecological problems in the Gan River, including water pollution, biodiversity decline, and habitat degradation (Feng et al., 2021; Guo et al., 2018; Yang et al., 2020). Samples were collected from 25 sampling sites (Figure 1; for details, see Table S1). The sampling time was from June to July (wet water period) and October to November (dry water period) in 2022.

### 2.2 Sampling methods

We used a 25‐μm mesh size net to collect phytoplankton qualitative samples, and these samples were preserved with an acid Lugol's iodine solution. Phytoplankton quantitative samples were collected using 1 L bottles and were also fixed using the acid Lugol's iodine solution. Quantitative samples were allowed to settle for 48 h and then concentrated to a final volume of 50 mL. A microscope (OLYMPUS CX21) was used to count phytoplankton cells. A wet weight of 1 g/cm^3^ was used to calculate the biomass. Species were identified to the possible taxonomic level (species or genus level).

### 2.3 Measurement of environmental factors

We used a Multi‐Parameter Probe (YSI, USA) to measure the water temperature (WT;°C), dissolved oxygen (DO; mg/L), hydrogen ions (pH), total dissolved solids (TDS; mg/L), salinity (Sal; mg/L), oxidation reduction potential (ORP; mv), electrical conductivity (EC; S/cm), and chlorophyll‐a (Chl‐a; mg/L). Transparency (SD; cm) was measured by using a Secchi disk. Water samples were refrigerated and transported to the Nanchang University laboratory. Total nitrogen (TN; mg/L) and total phosphorus (TP; mg/L), ammonia nitrogen (NH_3_‐N; mg/L), permanganate index (CODMn; mg/L), and copper (Cu) were analyzed using Ultraviolet Spectrophotometry.

### 2.4 Data analysis

Classification of phytoplankton functional groups was done according to Reynolds et al. (2002) and Padisak et al. (2009). The phytoplankton community was classified into 25 functional groups (for details, see Table S2): A, B, D, E, F, G, H1, J, LO, M, MP, N, NA, P, S1, S2, SN, T, TB, W1, W2, X1, X2, X3, and Y.

Shannon–Wiener diversity index (*H*′) was calculated to analyze the diversity of phytoplankton in the study area (Magurran, 1988; Peet, 1974). We used one‐way analysis of variance (ANOVA) performed in SPSS 22.0 to test for significant difference between number of species, density, biomass, diversity of phytoplankton, and environmental factors among study area and season (*p < .05; **p < .01; ***p < .001). The phytoplankton community structure was analyzed using the nonmetric multidimensional scaling (NMDS) ordination plots performed by PRIMER 6 (Clarke & Gorley, 2006). The relationship between the phytoplankton community structure and the environmental factors was analyzed using redundancy analysis (RDA) performed with CANOCO Version 5 (Ter Braak & Verdonschot, 1995).

The evaluation system of P‐IBI in the middle and lower reaches of the Gan River was constructed in three steps: selection and determination of reference and impaired sites, screening and determination of evaluation indicators, and quantification of evaluation indicators. First, by reviewing relevant literature studies (Cai et al., 2016; Kane et al., 2009; Zhu et al., 2021), following principles were applied to determine the reference and impaired sites in the middle and lower reaches of the Gan River: (1) the value of Shannon–Wiener diversity index was higher than two; (2) the water quality indicators in the sampling sites were higher than Class II (National Environmental Quality Standard for Surface Water, GB3838‐2002); (3) the phytoplankton density was less than 1,000,000 cells/L; and (4) the sampling sites were less affected by human activities. Second, we selected 27 candidate indicators for analyzing P‐IBI based on relevant literature studies (Cai et al., 2016; Zhang, et al., 2020; Huang et al., 2022; Qin et al., 2023; Table S3). Finally, candidate indicators were tested, and evaluation indicators of P‐IBI were determined and standardized. The P‐IBI score was calculated in the middle and lower reaches of the Gan River Basin and used to evaluate their aquatic ecological health.

## 3 RESULTS

### 3.1 Seasonal and spatial changes of taxonomic composition of phytoplankton

**FIGURE 2:**
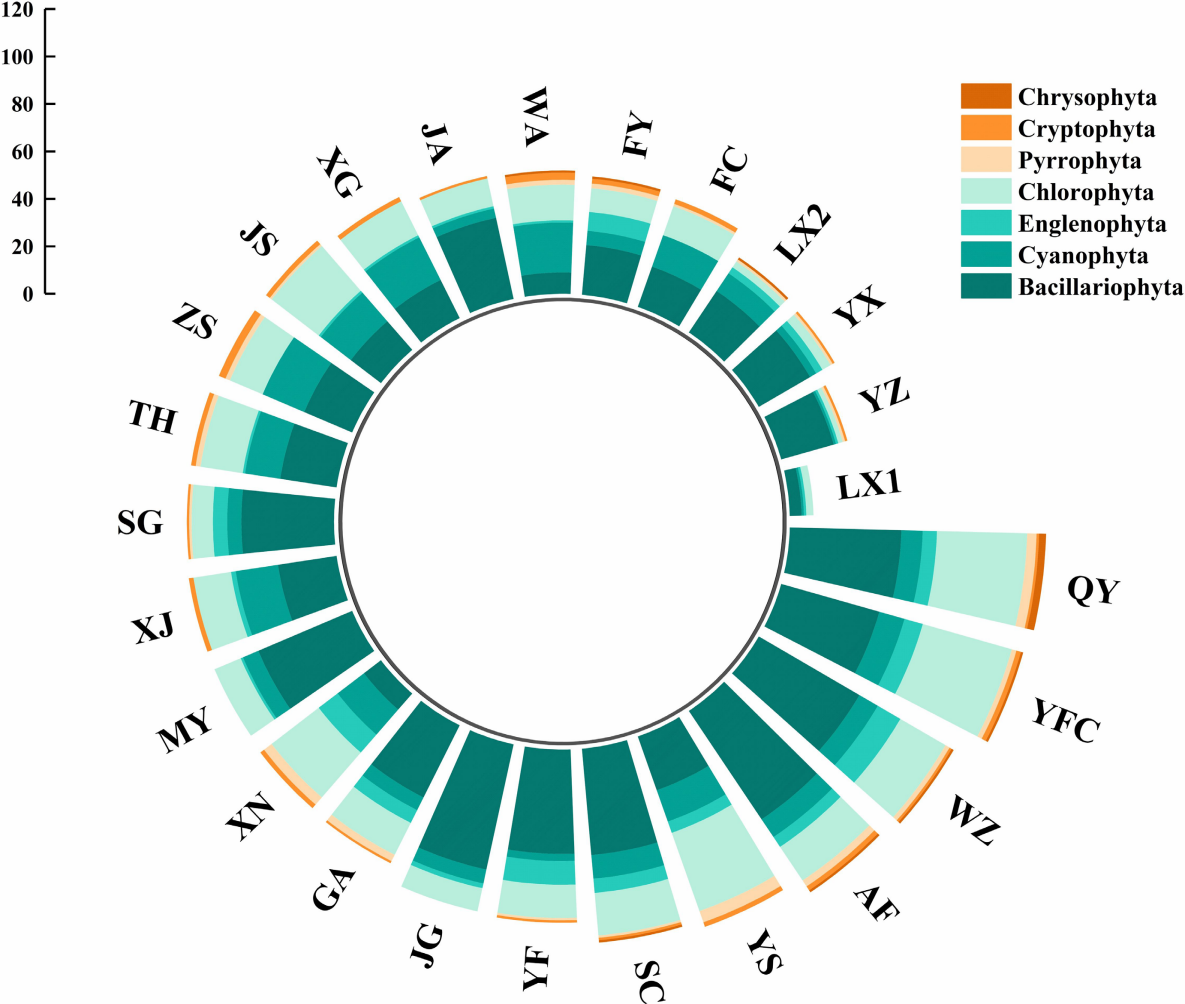
The composition of phytoplankton community in the middle and lower reaches of the Gan River. Sampling site codes are as in Table S1.

A total of 325 species of phytoplankton belonging to 7 phyla and 103 genera were identified in the middle and lower reaches of the Gan River. The number of Bacillariophyta species was the highest (115), followed by Chlorophyta (104), and the number of Cryptophyta species was the lowest (3). Significant seasonal (ANOVA, *F*_1,140_ = 5.82, *p* = .017) and spatial (ANOVA, *F*_24,117_ = 3.24, *p* = .001) differences were found among the number of phytoplankton species. The number of phytoplankton species in the wet period (279) was higher than that in the dry period (210) (Figure 2). The number of phytoplankton species in the QY (108) and YFC (106) were the highest, and the number of species in LX1 was the lowest (10) (Figure 2). The number of phytoplankton species in the tributaries of the Gan River (254 and 190) was higher than that in the main stream (119 and 94) during both the wet and dry periods, respectively.

### 3.2 Seasonal and spatial changes of phytoplankton density and biomass

The density of phytoplankton in the middle and lower reaches of the Gan River ranged from 30,000 to 39,050,000 cells/L, with an average density of 1,920,000 cells/L. The biomass of phytoplankton ranged from 0.01 to 7.41 mg/L, with an average biomass of 0.46 mg/L. Significant seasonal and spatial differences were found among the density (ANOVA, seasonal: *F*_1,140_ = 0.05, *p* = .823; spatial: *F*_24,117_ = 0.11, *p* = .736) and biomass of phytoplankton (ANOVA, seasonal: *F*_1,140_ = 125.12, *p* = .001; spatial: *F*_24,117_ = 19.40, *p* = .001). The density and biomass of phytoplankton in the wet period (224,000 cells/L; 0.53 mg/L; Figure 4a,b) were higher than those in the dry period (166,000 cells/L; 0.42 mg/L; Figure 4a,b). The density and biomass of the tributaries (259,000 cells/L, 0.63 mg/L; Figure 4a,b) were higher than those in the main stream (38,000 cells/L, 0.08 mg/L; Figure 4a,b).

### 3.3 Composition and biomass of phytoplankton functional groups

**FIGURE 3:**
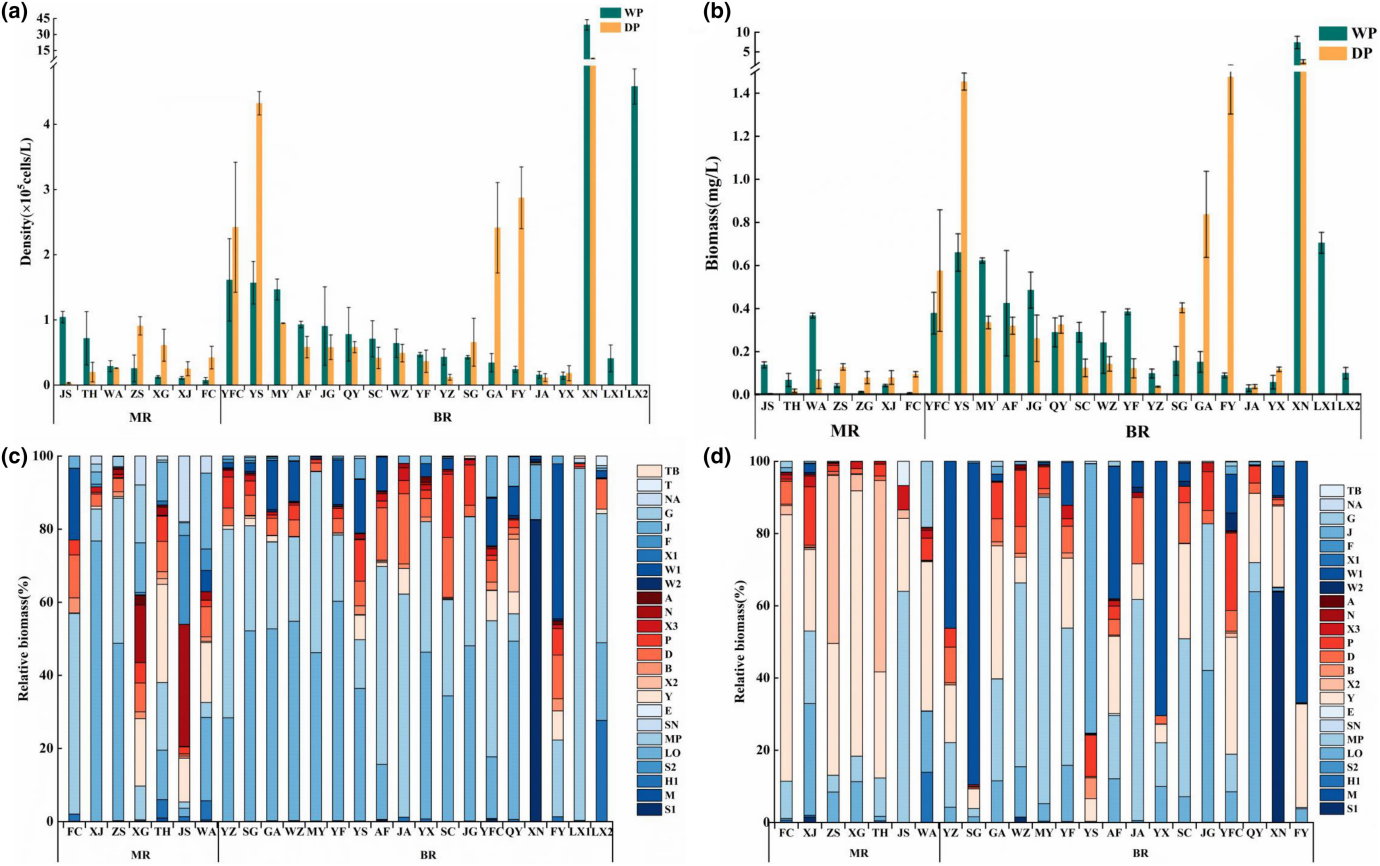
The seasonal and spatial differences of phytoplankton density (a) and biomass (b), and relative biomass of phytoplankton functional groups in wet (c) and dry (d) significant difference between phytoplankton diversity (*p < .05; **p < .01; ***p < .001) periods in the middle and lower reaches of the Gan River. Sampling site codes are as in Table S1. BR, Tributary of the Gan River; DP, Dry period; MR, Main stream of the Gan River; WP, Wet period.

The composition of phytoplankton functional group showed significant spatial (ANOVA, *F*_1,140_ = 1.63, *p* = .001) and seasonal (ANOVA, *F*_24,117_ = 3.89, *p* = .001) differences. The dominant functional groups were L_O_, MP, S1, W1, and Y (Figure 3c,d). The dominant functional groups in the wet period were S1, Lo, and MP and in the dry period were S1, L_O_, MP, Y, X2, W1, and J. The YFC had the greatest number of functional groups (22), and YX had the lowest number of functional groups (12) (Figure 3c,d). The dominant functional groups in the main stream were L_O_, MP, X2, and Y and in the tributaries were D, L_O_, MP, P, W1, and Y (Figure 3c,d).

### 3.4 Seasonal and spatial changes of phytoplankton community structure

**FIGURE 4:**
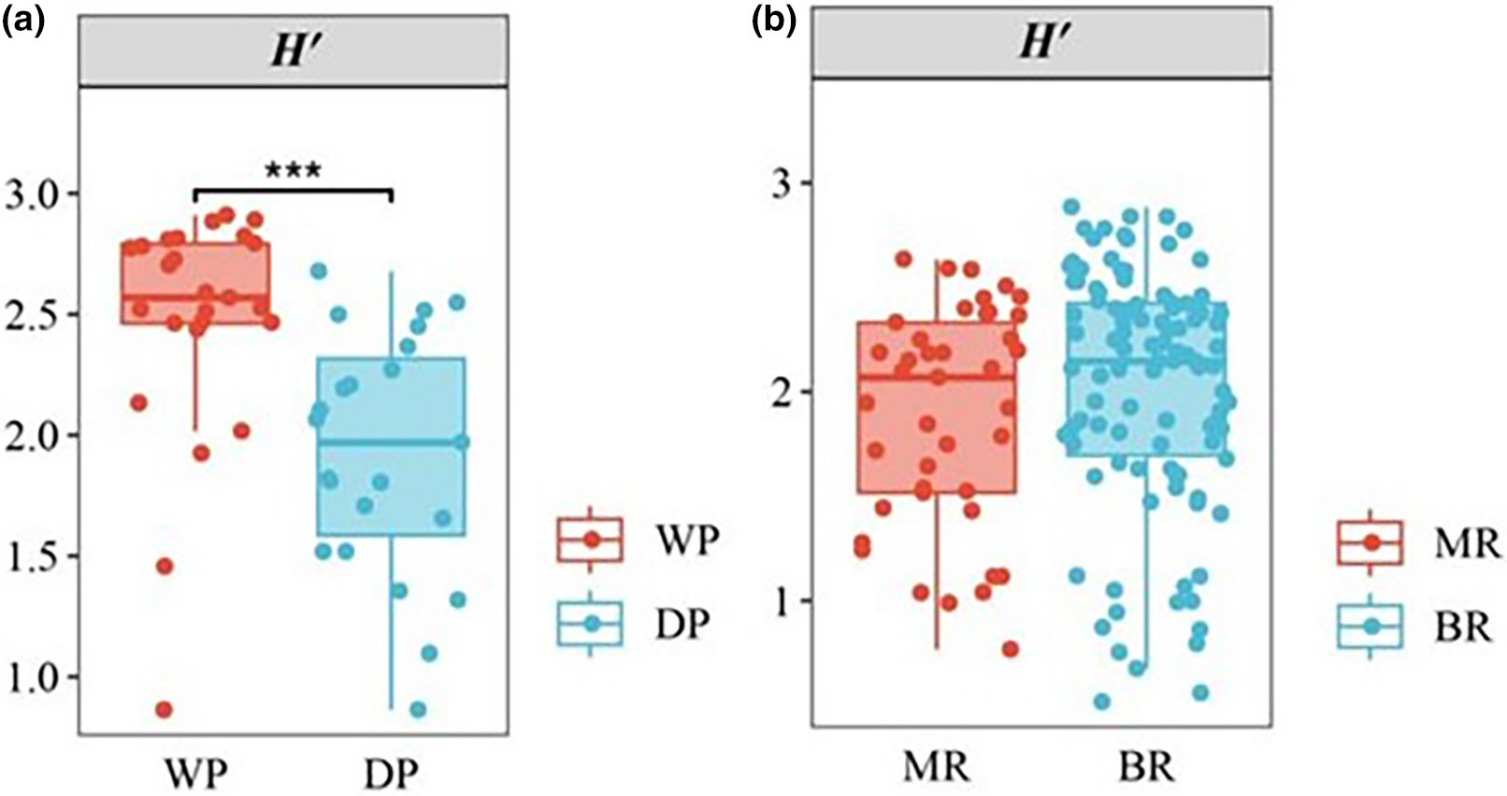
The seasonal (a) and spatial (b) differences of phytoplankton diversity in the middle and lower reaches of the Gan River. BR, Tributary of the Gan River; DP, Dry period; MR, Main stream of the Gan River; WP, Wet period. Significant difference between phytoplankton diversity (*p < .05; **p < .01; ***p < .001).

**FIGURE 5:**
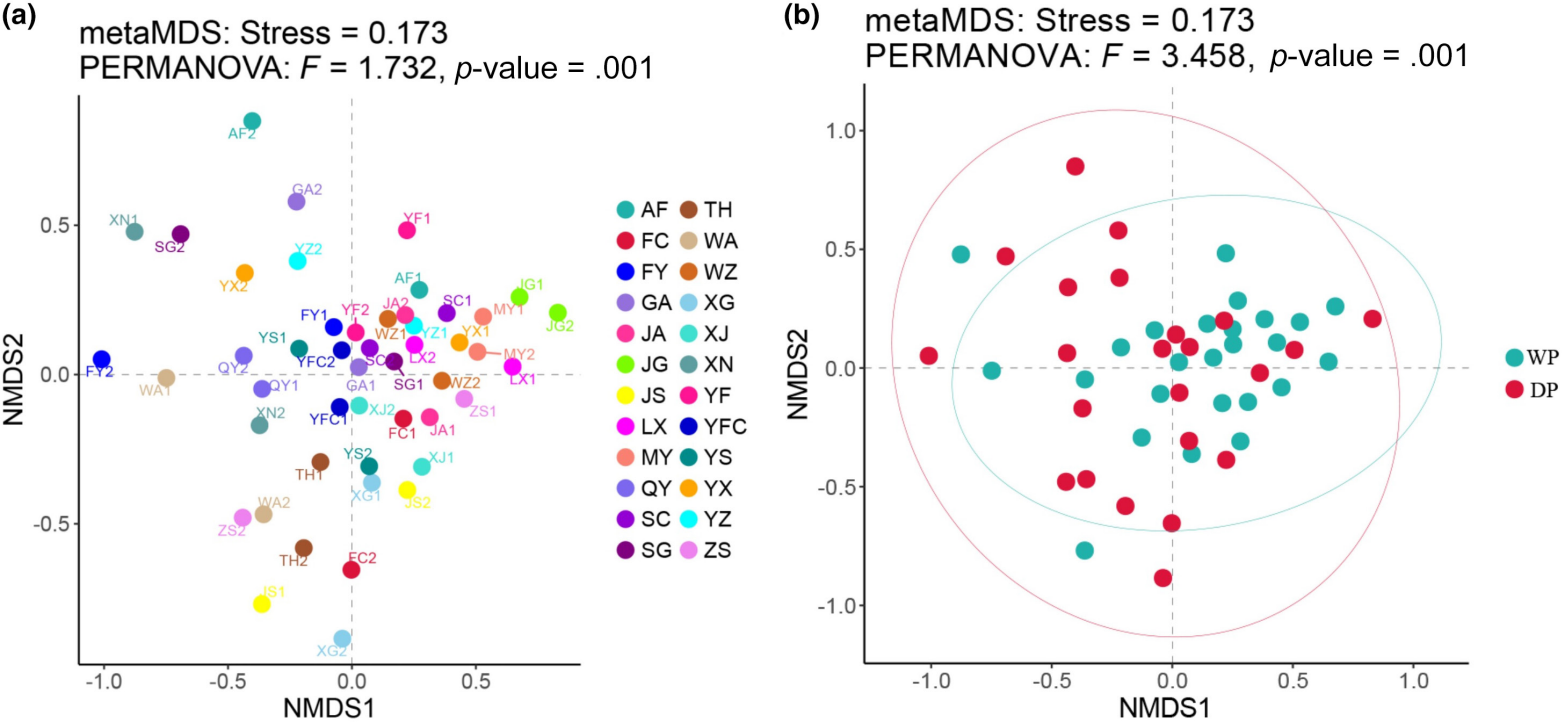
The nonmetric multidimensional scaling (NMDS) ordination in the seasonal (a) and spatial (b) differences of phytoplankton community structure in the middle and lower reaches of the Gan River. Sampling site codes are as in Table S1. DP, Dry period; WP, Wet period.

The Shannon–Wiener diversity index of phytoplankton ranged between 1.19 and 3.00. Significant seasonal and spatial differences were found among the phytoplankton diversity (ANOVA, *p* < .05; Figure 4). Phytoplankton diversity in the wet period was significantly higher than that in the dry period. The NMDS plot showed that the structure of phytoplankton community exhibited significant spatial (PERMANOVA, *F* = 1.732, *p* = .001; Figure 5a) and seasonal differences (PERMANOVA, *F* = 3.458, *p* = .001; Figure 5b).

### 3.5 Association between the phytoplankton community structure and the environmental factors

Water temperature (*F*_1,140_ = 402.31, *p* = .001), dissolved oxygen (*F*_1,140_ = 30.70, *p* = .001), pH(*F*_1,140_ = 9.15, *p* = .003), oxidation reduction potential (*F*_1,140_ = 22.22, *p* = .001), electrical conductivity (*F*_1,140_ = 10.18, *p* = .002), total dissolved solids (*F*_1,140_ = 3.97, *p* = .048), and salinity (*F*_1,140_ = 26.75, *p* = .001) showed significant seasonal differences (Table 1). Chlorophyll‐a (*F*_1,140_ = 0.01, *p* = .919), permanganate index (*F*_1,140_ = 0.03, *p* = .859), total nitrogen (*F*_1,140_ = 1.08, *p* = .310), total phosphorus (*F*_1,140_ = 1.20, *p* = .278), ammonia nitrogen (*F*_1,140_ = 1.58, *p* = .214), transparency (*F*_1,140_ = 0.11, *p* = .739), and copper (*F*_1,140_ = 0.95, *p* = .333) showed no significant seasonal differences (Table 1).

**TABLE 1:** Seasonal and spatial differences of environmental factors in wet and dry periods in the middle and lower reaches of the Gan River based on ANOVA analysis (mean ± SD).

	Wet period	Dry period	Spatial difference		Seasonal difference	
			*F*	*p*	*F*	*p*
WT (°C)	29.93 ± 3.91	21.13 ± 1.97	0.881	.619	402.314	.*001*
DO (mg/L)	7.81 ± 1.51	8.88 ± 1.01	2.567	.*001*	30.706	.*001*
TDS (mg/L)	0.18 ± 0.08	0.27 ± 0.12	1.206	.259	3.977	.*048*
Sal (%)	0.13 ± 0.06	0.21 ± 0.11	6.064	*.001*	26.758	.*001*
pH	7.68 ± 0.42	7.87 ± 0.38	3.645	.*001*	9.156	.*003*
ORP (mV)	13.40 ± 26.38	40.84 ± 30.50	1.320	.176	22.220	.*001*
EC (μS/cm)	304.41 ± 141.70	413.76 ± 197.10	8.415	*.001*	10.184	.*002*
Chl‐*a* (μg/L)	1.21 ± 0.85	1.24 ± 1.56	8.599	.*001*	0.010	.919
COD_Mn_ (mg/L)	2.20 ± 0.58	2.38 ± 0.76	1.429	.195	0.032	.859
TN (mg/L)	0.95 ± 0.35	1.00 ± 0.32	2.486	.084	1.088	.310
TP (mg/L)	0.05 ± 0.03	0.05 ± 0.03	1.490	.169	1.208	.278
NH_3_‐N (mg/L)	0.20 ± 0.11	0.93 ± 0.40	0.875	.625	1.588	.214
SD (m)	1.40 ± 0.65	1.43 ± 0.60	2.536	.053	0.112	.739
Cu (mg/L)	0.0014 ± 0.0012	0.0015 ± 0.0012	2.809	*.007*	0.957	.333

*Note:* Italic value of *p* indicated that environmental factors had spatial and seasonal differences.

Abbreviations: Chl‐a, Chlorophyll‐a; COD_Mn_, Permanganate index; Cu, Copper; DO, Dissolved oxygen; EC, Electrical conductivity; NH_3_‐N, Ammonia nitrogen; ORP, Oxidation reduction potential; pH, Hydrogen ions; Sal, Salinity; SD, Transparency; TDS, Total dissolved solids; TN, Total nitrogen; TP, Total phosphorus; WT, Water temperature.

Dissolved oxygen (*F*_24,117_ = 2.56, *p* = .001), electrical conductivity (*F*_24,117_ = 8.41, *p* = .001), salinity (*F*_24,117_ = 6.06, *p* = .001), pH(*F*_24,117_ = 3.64, *p* = .001), chlorophyll‐a (*F*_24,117_ = 8.59, *p* = .001), and copper (*F*_24,117_ = 2.80, *p* = .007) showed significant spatial difference (Table 1). Water temperature (*F*_24,117_ = 0.88, *p* = .619), oxidation reduction potential (*F*_24,117_ = 1.32, *p* = .176), TDS(*F*_24,117_ = 1.20, *p* = .259), total nitrogen (*F*_24,117_ = 2.48, *p* = .084), total phosphorus (*F*_24,117_ = 1.49, *p* = .169), permanganate index (*F*_24,117_ = 1.42, *p* = .195), transparency (*F*_24,117_ = 2.53, *p* = .053) and ammonia nitrogen (*F*_24,117_ = 0.87, *p* = .625) showed no significant spatial differences (Table 1).

**FIGURE 6:**
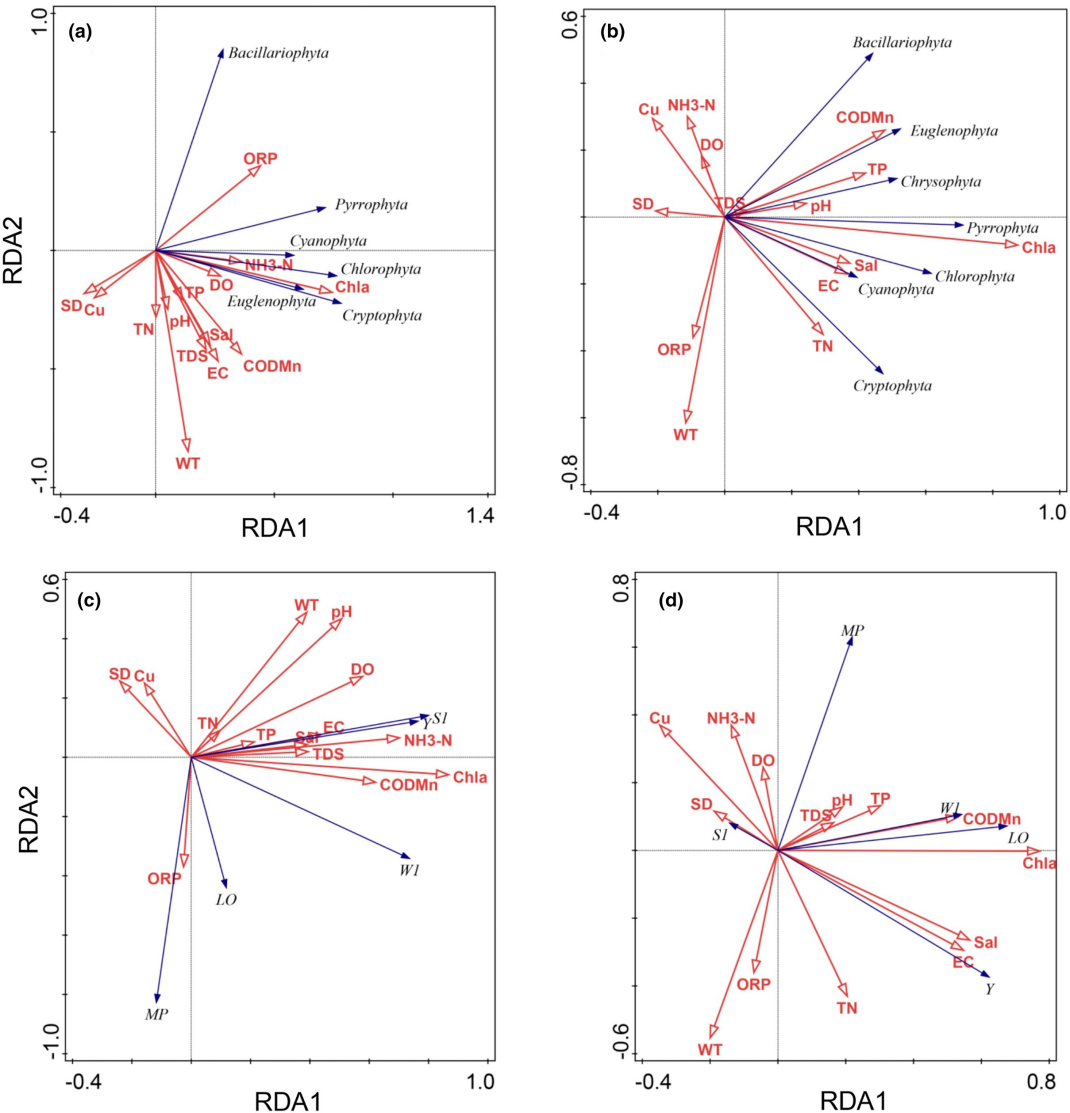
Ordination biplot of phytoplankton taxonomic groups in the wet (a) and dry (b) periods and functional groups in the wet (c) and dry (d) periods and environmental variables based on RDA in the middle and lower reaches of the Gan River. Chl‐a, Chlorophyll‐a; COD_Mn_, Permanganate index; Cu, Copper; DO, Dissolved oxygen; EC, Electrical conductivity; NH_3_‐N, Ammonia nitrogen; ORP, Oxidation reduction potential; pH, Hydrogen ions; Sal, Salinity; SD, Transparency; TDS, Total dissolved solids; TN, Total nitrogen; TP, Total phosphorus; WT, Water temperature.

The eigenvalues of taxonomic groups along the first axis in the wet and dry periods were 0.42 and 0.29, respectively. The species–environment cumulative variances along the first axis were 57.53% and 52.46%, respectively. Chlorophyll‐a, water temperature, pH, and oxidation reduction potential were significantly correlated with the taxonomic groups in the wet period (Figure 6a). Chlorophyll‐a was significantly positively correlated with Cyanophyta, Chlorophyta, Cryptophyta, Pyrrophyta, and Euglenophyta and was significantly negatively correlated with Bacillariophyta (Figure 6a). The Cyanophyta, Chlorophyta, Cryptophyta, and Euglenophyta showed a significant positive correlation with water temperature and pH, whereas Bacillariophyta and Pyrrophyta exhibited a significant negative correlation (Figure 6a). Chlorophyll‐a, ammonia nitrogen, total nitrogen, transparency, electrical conductivity, copper, and permanganate index were significantly correlated with the taxonomic groups in the dry period (Figure 6b). Chlorophyll‐a was significantly positively correlated with all phyla (Figure 6b).

The eigenvalues of functional groups along the first axis in the wet and dry periods were 0.36 and 0.24, respectively. The species–environment cumulative variances along the first axis were 47.32% and 44.30%, respectively. Chlorophyll‐a, water temperature, and pH were significantly correlated with the dominant functional groups in the wet period (Figure 6c). Chlorophyll‐a was significantly negatively correlated with MP and was significantly positively correlated with other dominant functional groups (Figure 6c). Water temperature and pH were significantly positively correlated with W1, Y, and S1 and were significantly negatively correlated with Lo and MP (Figure 6c). Chlorophyll‐a, water temperature, ammonia nitrogen, total phosphorus, transparency, copper, and permanganate index were significantly correlated with the dominant functional groups in the dry period (Figure 6d). Chlorophyll‐a was significantly positively correlated with MP, Y, LO, and W1 and was significantly negatively correlated with S1 (Figure 6d).

### 3.6 Ecological evaluation of the Gan River basin

**FIGURE 7:**
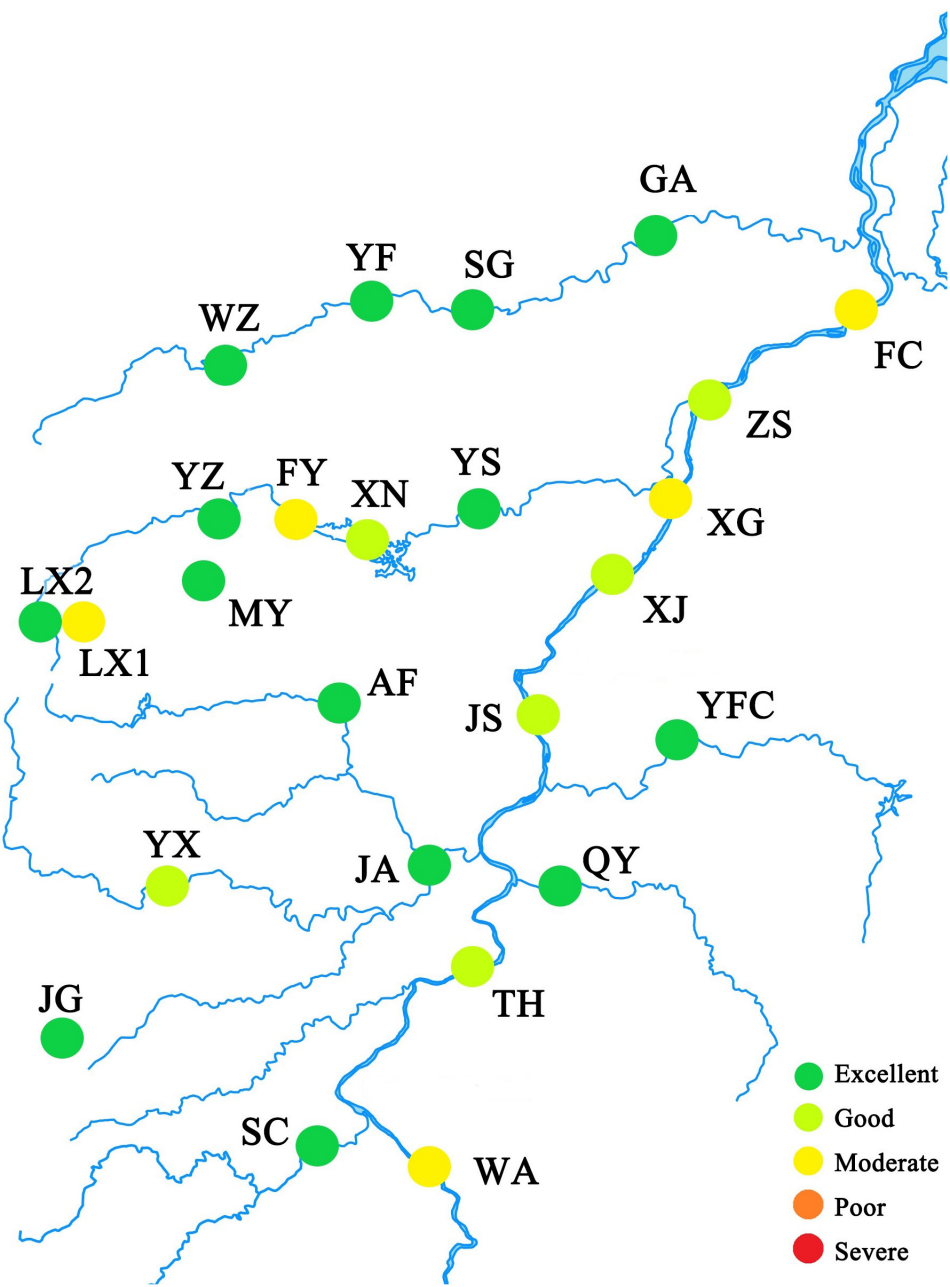
The phytoplankton biological integrity index (P‐IBI) in the middle and lower reaches of the Gan River. Sampling site codes are as in Table S1.

The P‐IBI of the middle and lower reaches of the Gan River was evaluated as excellent, the main stream was evaluated as moderate, and the tributaries were evaluated as excellent (Figure 7).

## 4 DISCUSSION

The Yangtze River Basin is one of the most heavily impacted basins by anthropogenic activities and faces significant challenges in biodiversity conservation (Liu et al., 2019; Zhang et al., 2020). To improve the integrity of the freshwater ecosystem, China imposed the TYFB in the Yangtze River Basin. In this study, the seasonal and spatial phytoplankton variations and association of environmental factors were analyzed in the middle and lower reaches of the Gan River to document the changes associated with the TYFB. Our results indicated an increase in the number of phytoplankton species from 312 before the TYFB to 325 after the TYFB. The dominant taxa of phytoplankton were both Chlorophyta and Bacillariophyta before and after the TYFB. Phytoplankton diversity ranged from 2.00 to 3.00 between 2008 and 2011; 0.15 to 1.83 in 2016; 1.00 to 2.00 in 2020; and 1.19 to 3.00 in this study, indicating an increase in diversity after the TYFB. In the 1980s, phytoplankton density and biomass were 0.59 × 10^5^ ind./L (Chen et al., 2009; Hu & Lin, 1988; Ji et al., 2012; Liu et al., 2012) and 0.26 mg/L (Chen et al., 2009; Hu & Lin, 1988; Ji et al., 2012; Liu et al., 2012), respectively. By 2016, these values had increased to 31.5 × 10^5^ ind./L (Feng et al., 2021; Yang et al., 2020) and 0.69 mg/L (Feng et al., 2021; Yang et al., 2020), but the results of this study show a reduction to 1.92 × 10^5^ ind./L in density and 0.46 mg/L in biomass, suggesting a decline in both metrics following the TYFB. The decline could be attributed to the significantly reduced fishing pressure from the imposition of the TYFB, which may have increased the abundance of planktivorous fish and thus regulated plankton communities. To address this, further research that includes an analysis of fish communities is necessary.

There were significant seasonal and spatial differences in phytoplankton community structure, with environmental factors being important in driving these changes. The P‐IBI of the Gan River was evaluated as excellent after the TYFB. In the 1980s, water quality in the Gan River was classified as mildly polluted (Hu & Lin, 1988). By 2016, it had deteriorated to between moderate and heavy pollution. Results from this study, however, indicate a reduction to mild to moderate pollution based on P‐IBI, suggesting an improvement since the TYFB (Feng et al., 2021; Yang et al., 2020). These findings highlight that the implementation of TYFB supports efforts in biodiversity conservation and ecosystem restoration.

### 4.1 Temporal and spatial changes of phytoplankton community structure

There were a significant seasonal and spatial differences among the structure of phytoplankton community. The species number, density, biomass, and diversity of phytoplankton in the wet period were higher than those in the dry period, which may be attributed to the river flows and physical disturbances, nutrient changes, macrophyte structure, and hydrological alterations (Butler et al., 2007; Liu et al., 2020; Navas‐Parejo et al., 2020; Qin et al., 2020; Yang et al., 2017). For example, the river habitats in different seasons became more complex because of macrophyte structure, which played an important role in regulating the phytoplankton community structure (Gross et al., 2007; Ibelings et al., 2007). The nutrient concentrations in the seasonal and spatial changes were different, and therefore, nutrients played a critical role in community dynamics by supporting phytoplankton growth (Muhid et al., 2013; Navas‐Parejo et al., 2020). Hydrological alterations can lead to seasonal and spatial changes of aquatic organisms, which affects the phytoplankton community structure (Butler et al., 2007; Yang et al., 2017). River flows and physical disturbances affected phytoplankton washout and density‐independent mortality of many species, which played a critical role in community dynamics by changing phytoplankton community composition (Cook et al., 2010; Townsend & Douglas, 2017).

### 4.2 Key environmental factors of driver phytoplankton community structure

Chlorophyll‐a, pH, permanganate index, ammonia nitrogen, total phosphorus, transparency, water temperature, and copper were important environmental factors affecting the phytoplankton community structure in the Gan River Basin. Chlorophyll‐a and water temperature significantly affected the phytoplankton community structure. Chlorophyll‐a is related to the density or biomass of phytoplankton and significantly affects the number of functional groups under different hydrological conditions (Su et al., 2023; Sun et al., 2023). Water temperature is a key environmental factor influencing the phytoplankton community structure (Gong et al., 2023; Pan et al., 2014). In this study, water temperature showed significant positive correlation with S1, Y, and W1, promoting the growth of Cyanophyta, Cryptophyta, and Euglenophyta, while it showed significant negative correlation with LO and MP, inhibiting the growth of Bacillariophyta. Ammonia nitrogen showed a significant positive correlation with S1 and MP, which was consistent with the high nitrogen suitability of the Bacillariophyta and Cyanophyta (Cao et al., 2022; Ou et al., 2014; Wu et al., 2013). Bacillariophyta and Cyanophyta can make use of dissolved organic nitrogen directly, adapt to turbid mixed water, and often distributed in higher concentrations of dissolved or suspended matter (Wu et al., 2014). The N/P ratio changes the growth and reproduction of phytoplankton and has a great influence on the phytoplankton community structure (Schindler et al., 2008). Phytoplankton are phosphorus‐limited when the N/P atomic ratio is >20:1, and nitrogen‐limited when it is <10:1 (Schanz & Juon, 1983). The N/P ratio in the Gan River was 20:1, indicating that it was phosphorus‐limited. Total phosphorus was positively correlated with MP, L_O_, W1, and Y in the dry period, which were mostly adapted to the eutrophic water environment (Lv et al., 2023). Permanganate index was also an important environmental factor affecting the phytoplankton community structure. Permanganate index reflects the content of organic matter in water habitats and affects the growth and metabolism of phytoplankton (Han & Fan, 2012; Lan, 2018). Permanganate index in this study was positively correlated with most of the functional groups.

## 5 CONCLUSION

The present study revealed the changes in phytoplankton community structure associated with the TYFB. Based on the research findings, TYFB resulted in the increase in phytoplankton species, increase in species diversity, and decrease in phytoplankton biomass and densities. Seasonal changes, spatial patterns, and environmental changes were the main driving factors of the phytoplankton community structure. The health status of aquatic ecosystem showed a tendency to improve after the TYFB. These results indicated that the TYFB played an important role in the restoration of the studied freshwater ecosystem. Future studies should focus on the dynamics of fish, zooplankton, and macroinvertebrate community structures and their implications for phytoplankton communities in the river ecosystem to better assess the impact of the TYFB.

## AUTHOR CONTRIBUTIONS

**Peng Ye:** Formal analysis (equal); investigation (equal); methodology (equal); resources (equal); software (equal); writing – original draft (equal). **Xinwen Lu:** Formal analysis (equal); investigation (equal); methodology (equal). **Wenxin Xia:** Formal analysis (equal); investigation (equal); methodology (equal). **Yating Wang:** Formal analysis (equal); investigation (equal); methodology (equal). **Chunhua Zhou:** Formal analysis (equal); funding acquisition (equal); investigation (equal); methodology (equal). **Xiongjun Liu:** Data curation (equal); formal analysis (equal); software (equal); writing – original draft (equal); writing – review and editing (equal). **Shan Ouyang:** Data curation (equal); formal analysis (equal); investigation (equal); methodology (equal); software (equal); writing – original draft (equal); writing – review and editing (equal). **Xiaoping Wu:** Conceptualization (equal); formal analysis (equal); funding acquisition (equal); validation (equal); writing – original draft (equal); writing – review and editing (equal).

## CONFLICT OF INTEREST STATEMENT

None declared.

## Supporting information


Table S1.

Table S2.

Table S3.

Table S4.

Table S5.

Table S6.

## Data Availability

The data used in this manuscript were obtained from field investigations and laboratory experiments (taxon composition). The authors have provided the taxon information in supplemental files. Please see Table S4, Table S5, and Table S6.
