# Supplementary material for: Effect of the ten‐year fishing ban on change of phytoplankton community structure: Insights from the Gan River
Source: Ecol Evol. 2024 Aug 29;14(9):e70217. doi: 10.1002/ece3.70217 (PMC11362611; doi:10.1002/ece3.70217)
Supplement: Supplementary file 1 — Table S1. [file ECE3-14-e70217-s003.docx]

**Table S1** Sampling time and location of sampling site in the middle and lower reaches of the Gan River

| Sampling sites | Code | Longitude | Latitude | Sampling time |
| --- | --- | --- | --- | --- |
| Zhangshu City | ZS | 115.4957 | 28.0422 | July and October in 2022 |
| Wan 'an County | WA | 114.8750 | 26.3793 | August and October in 2022 |
| Jishui County | JS | 115.1208 | 27.3523 | August and October in 2022 |
| Xiajiang County | XJ | 115.2921 | 27.6643 | August and October in 2022 |
| Xingan County | XG | 115.4084 | 27.8202 | August and October in 2022 |
| Fengcheng City | FC | 115.8168 | 28.2357 | August and October in 2022 |
| Taihe County | TH | 114.9751 | 26.8105 | August and November in 2022 |
| Suichuan County | SC | 114.6322 | 26.4185 | August and October in 2022 |
| Yongxin County | YX | 114.3104 | 27.0059 | August and October in 2022 |
| Ji 'an County | JA | 114.8686 | 27.0186 | August and October in 2022 |
| Qingyuan District | QY | 115.1653 | 26.9900 | August and November in 2022 |
| Yongfeng County | YFC | 115.4208 | 27.2997 | August and November in 2022 |
| Anfu County | AF | 114.6721 | 27.3946 | August and October in 2022 |
| Jinggang Mountain | JG | 114.0675 | 26.6621 | August and October in 2022 |
| Mingyue Mountain | MY | 114.3779 | 27.6371 | July and November in 2022 |
| Yuanzhou District | YZ | 114.4106 | 27.7967 | July and November in 2022 |
| Wanzai County | WZ | 114.4308 | 28.1142 | July and November in 2022 |
| Shanggao County | SG | 114.9837 | 28.2481 | July and November in 2022 |
| Yifeng County | YF | 114.7501 | 28.2573 | July and November in 2022 |
| Gao 'an City | GA | 115.3576 | 28.4096 | July and November in 2022 |
| Fenyi County | FY | 114.5856 | 27.7933 | July and November in 2022 |
| Yushui District | YS | 114.9761 | 27.8088 | July and November in 2022 |
| Xiannv Lake | XN | 114.7515 | 27.7397 | July and November in 2022 |
| Luxi No.1 | LX1 | 114.1041 | 27.5636 | June in 2022 |
| Luxi No.2 | LX2 | 114.0321 | 27.5617 | June in 2022 |
