# Supplementary material for: Effect of the ten‐year fishing ban on change of phytoplankton community structure: Insights from the Gan River
Source: Ecol Evol. 2024 Aug 29;14(9):e70217. doi: 10.1002/ece3.70217 (PMC11362611; doi:10.1002/ece3.70217)
Supplement: Supplementary file 2 — Table S2. [file ECE3-14-e70217-s002.docx]

Table S2 Composition of phytoplankton functional groups in the middle and lower reaches of Gan River

| Code | Typical representatives | Habitat template |
| --- | --- | --- |
| A | *Diatoma* sp., *Attheya* *zachariasi* | clear, deep, base poor lakes, with species sensitive to pH rise |
| B | *Cyclotella* sp. | mesotrophic small- and medium-sized lakes with species sensitive to the onset of stratification |
| D | *Nitzschia* sp., *Synedra* *acus*, *Synedra* *ulna*, *Stauroneis* *anceps* | shallow turbid waters including rivers |
| E | *Dinobryon* sp. | usually small, shallow, base poor lakes or heterotrophic ponds |
| F | *Oocystis* sp., *Dictyosphaerium* *pulchellum*, *Treubaria* *triappendiculata*, *Quadrigula* *chodatii*, *Palmellococcus* *miniatus* | clear, deeply mixed meso-eutrophic lakes |
| G | *Pandorina* *morum*, *Eudorina* *elegans* | nutrient-rich conditions in stagnating water columns; small eutrophic lakes and very stable phases in larger river-fed basins and storage reservoirs |
| H1 | *Annabaena* spp. | eutrophic, both stratified and shallow lakes with low nitrogen content |
| J | *Tetraëdron* *minimum*, *Scenedesmus* spp., *Crucgenia* sp., *Pediastrum* sp., *Coelastrum* sp., *Actinastrum* sp., *Golenkinia* sp. | shallow, mixed, highly enriched systems |
| L_O_ | *Merismopedia* sp., *Chroococcus* *limneticus*, *Chroococcus* *minutus*, *Coelosphaerium* *dubium*, *Peridinium* sp., *Ceratium* *hirundinella*, *Pinnularia* sp., *Amphora* *ovalis* | deep and shallow, oligo to eutrophic, medium to large lakes |
| M | *Microcystis* sp. | eutrophic to hypertrophic, small- to medium-sized water bodies |
| MP | *Oscillatoria* sp., *Cocconeis* *placentula*, *Navicula* sp., *Gomphomema* sp., *Cymbella* sp., *Surirella* sp., *Achnanthes* sp., *Eunotia* sp., *Ulothrix* *zonata* | frequently stirred up, inorganically turbid shallow lakes |
| N | *Tabellaria* sp., *Cosnarium* sp., *Staurodesmus* sp. | continuous or semi-continuous mixed layer of 2–3 m in thickness |
| N_A_ | *Staurastrum* sp. | oligo-mesotrophic, atelomictic environments at lower latitudes with species sensitive to destratification |
| P | *Fragilaria* sp., *Melosira* *granulata*, *Melosira* *granulata* *var*. *angustissima*, *Closterium* sp. | similar to that of codon N but at higher trophic states |
| S1 | *Leptolyngbya* spp. | turbid mixed environments |
| S2 | *Spirulina* *major* | warm, shallow and often highly alkaline waters |
| S_N_ | *Raphidiopsis* *sinensia* | warm mixed environments |
| T | *Planctonema* *lauterbornii* | persistently mixed layers, in which light is increasingly the limiting constraint and thus optically deep, mixed environments including clear epilimnia of deep lakes in summer |
| T_B_ | *Melosira* *varians*, *Raphidonema* sp. | highly lotic environments (streams and rivulets) |
| W1 | *Euglena* spp., *Phacus* sp. | ponds, even temporary, rich in organic matter from husbandry or sewages |
| W2 | *Trachelomonas* sp., *Strombomonas* *verrucosa* | meso-eutrophic ponds, even temporary, shallow lakes |
| X1 | *Chlorella* *vulgaris*, *Ankistrodesmus* sp., *Schroederia* sp. | shallow, eu-hypertrophic environments |
| X2 | *Cryptomonas* *acuta*, *Chlamydomonas* sp., *Pteromonas* sp. | shallow, meso-eutrophic environments |
| X3 | *Gyrosigma* sp., *Cymatopleura* sp., *Neidium* sp., *Characium* *limneticum* | shallow, well mixed oligotrophic environments |
| Y | *Cryptomonas* *ovata*, *Cryptomonas* *erosa*, *Glenodinium* sp. | this codon, mostly including large cryptomonads but also small dinoflagellates, refers to a wide range of habitats, which reflect the ability of its representative species to live in almost all lentic ecosystems when grazing pressure is low |
