# Supplementary material for: Effect of the ten‐year fishing ban on change of phytoplankton community structure: Insights from the Gan River
Source: Ecol Evol. 2024 Aug 29;14(9):e70217. doi: 10.1002/ece3.70217 (PMC11362611; doi:10.1002/ece3.70217)
Supplement: Supplementary file 3 — Table S3. [file ECE3-14-e70217-s006.docx]

Table S3 Candidate indicators of phytoplankton biological integrity index

| Type of  indicator | Candidate indicators | | Response to  interference | | Code | |
| --- | --- | --- | --- | --- | --- | --- |
| Species  composition | Total number of taxa | | Decreased | | M1 | |
|  | Bacillariophyta taxa | | Decreased | | M2 | |
|  | Chlorophyta taxa | Decreased | | M3 | |  |
|  | Cyanophyta taxa | Decreased | | M4 | |  |
|  | Bacillariophyta taxa (%) | Decreased | | M5 | |  |
|  | Chlorophyta taxa (%) | Decreased | | M6 | |  |
|  | Cyanophyta taxa (%) | Decreased | | M7 | |  |
| Density | Total density | Increased | | M8 | |  |
|  | Bacillariophyta density | Decreased | | M9 | |  |
|  | Chlorophyta density | Decreased | | M10 | |  |
|  | Cyanophyta density | Decreased | | M11 | |  |
|  | Bacillariophyta density (%) | Decreased | | M12 | |  |
|  | Chlorophyta density (%) | Increased | | M13 | |  |
|  | Cyanophyta density (%) | Increased | | M14 | |  |
| Biomass | Total biomass | Increased | | M15 | |  |
|  | Cyanophyta biomass | Increased | | M16 | |  |
|  | Bacillariophyta biomass | Increased | | M17 | |  |
|  | Chlorophyta biomass | Increased | | M18 | |  |
|  | Cyanophyta biomass (%) | Increased | | M19 | |  |
|  | Bacillariophyta biomass (%) | Increased | | M20 | |  |
|  | Chlorophyta biomass (%) | Increased | | M21 | |  |
| Diversity  index | *H′* | Decreased | | M22 | |  |
|  | *J′* | Decreased | | M23 | |  |
|  | d_Ma_ | Decreased | | M24 | |  |
|  | *D* | Decreased | | M25 | |  |
| Dominant  species | Top three dominant species density (%) | Increased | | M26 | |  |
| Nutritional  structure | Diatom quotient | Increased | | M27 | |  |
