# Supplementary material for: Effect of the ten‐year fishing ban on change of phytoplankton community structure: Insights from the Gan River
Source: Ecol Evol. 2024 Aug 29;14(9):e70217. doi: 10.1002/ece3.70217 (PMC11362611; doi:10.1002/ece3.70217)
Supplement: Supplementary file 4 — Table S4. [file ECE3-14-e70217-s001.docx]

**Table S4** Distribution and composition of phytoplankton in the middle and lower reaches of the Gan River; Sampling site codes are as in Table S1. WP: wet period; DP: dry period

|  | MR | | | | | | | BR | | | | | | | | | | | | | | | | | |
| --- | --- | --- | --- | --- | --- | --- | --- | --- | --- | --- | --- | --- | --- | --- | --- | --- | --- | --- | --- | --- | --- | --- | --- | --- | --- |
|  | ZS | FC | WA | TH | JS | XJ | XG | SC | YX | JA | QY | YFC | AF | JG | MY | YZ | WZ | SG | YF | GA | FY | YS | XN | LX1 | LX2 |
| *Cocconeis placentula* | + |  | + | + | + | + |  | + | + | + |  |  | + | + | + | + | + | + | + | + | + | + |  | + | + |
| *Attheya zachariasi* |  |  | + | + | + | + | + |  |  |  |  |  | + |  |  |  |  |  |  |  |  | + |  |  |  |
| *Achnanthes linearis* |  |  |  |  |  |  |  |  |  |  |  |  |  |  |  |  |  | + |  |  |  |  |  |  |  |
| *Achnanthes crenulata* |  |  |  |  |  |  |  |  |  |  |  |  |  | + |  |  |  |  |  |  |  |  |  |  |  |
| *Achnanthes* sp. |  |  |  |  |  |  |  |  |  |  |  |  |  |  | + |  |  |  |  |  |  |  |  |  |  |
| *Stephanodiscus asteaea* | + |  |  |  |  |  |  |  |  |  | + |  |  | + |  |  |  |  |  | + |  |  |  |  |  |
| *Neidium kozlowi var. parva* |  |  |  |  |  |  |  |  |  |  |  |  |  |  |  |  |  | + |  |  |  |  |  |  |  |
| *Neidium dubium* |  |  |  |  |  |  |  |  |  |  |  |  |  |  |  |  | + |  |  |  |  |  |  |  |  |
| *Neidium* sp. |  |  |  |  |  | + |  |  |  |  |  |  |  |  |  |  |  | + |  |  |  |  |  |  |  |
| *Cymbella laevis* |  |  |  |  |  |  |  |  |  |  |  |  | + |  |  |  | + |  |  |  |  |  |  |  | + |
| *Cymbella cistula* |  |  |  |  |  |  |  |  |  |  |  |  |  | + |  |  |  |  |  |  |  |  |  |  |  |
| *Cymbella pusilla* |  |  |  |  | + | + |  |  |  | + |  |  |  | + |  |  | + |  |  |  |  |  |  |  |  |
| *Cymbella affinis* |  |  |  |  |  |  |  | + |  | + | + |  |  | + |  |  |  | + |  | + | + |  |  |  |  |
| *Cymbella ehrenbergii* |  | + |  | + |  |  |  | + | + | + | + |  | + |  | + |  | + | + | + | + |  |  |  |  |  |
| *Cymbella tumida* |  |  |  |  |  |  |  | + | + | + | + | + | + | + | + |  | + | + | + | + |  | + |  |  | + |
| *Cymbella ventricosa* |  |  |  |  |  |  |  |  |  |  |  |  |  |  | + |  |  |  |  |  |  |  |  |  |  |
| *Cymbella austriaca* |  |  |  |  |  |  |  |  |  | + |  |  |  |  |  |  |  |  |  |  |  |  | + |  |  |
| *Cymbella cymbiformis* |  |  |  |  |  |  |  |  |  |  |  |  |  |  |  |  | + |  |  |  |  |  |  |  |  |
| *Cymbella* sp. | + | + |  |  |  | + | + | + | + | + | + | + | + | + |  | + | + | + | + |  | + |  |  |  |  |
| *Diatoma* sp. |  |  |  |  |  |  | + | + |  | + | + |  | + |  |  |  | + | + | + |  |  |  |  |  |  |
| *Amphora ovalis* | + |  |  |  |  |  |  |  |  |  | + |  | + | + |  |  | + | + | + | + |  |  |  |  |  |
| *Amphora* sp. |  | + |  |  |  |  | + | + | + | + |  | + | + | + |  |  | + | + | + |  |  |  |  |  |  |
| *Hantzschia amphioxys* |  |  |  |  |  |  |  |  |  |  |  |  |  | + | + |  |  |  |  |  |  |  |  |  | + |
| *Hantzschia amphioxys f.capitata* | + |  |  |  |  |  |  | + |  |  |  |  |  |  |  |  |  |  |  |  |  |  |  |  |  |
| *Hantzschia elongata* |  |  |  |  |  |  |  |  |  |  |  |  | + |  |  |  |  |  |  |  |  |  |  |  |  |
| *Frustulia viridula* |  |  |  |  |  |  |  |  |  |  |  |  |  |  |  |  | + |  |  |  |  |  |  |  |  |
| *Stauroneis anceps f. linearis* | + |  |  | + |  |  |  | + |  |  | + | + |  | + | + |  | + | + | + | + |  |  |  |  |  |
| *Stauroneis anceps* |  |  |  | + | + | + |  | + | + | + | + | + | + | + | + | + | + | + | + | + | + | + | + |  | + |
| *Comphonema angustatum var. productum* |  |  |  |  |  |  |  |  |  |  | + |  |  | + | + |  | + |  |  | + |  |  |  |  |  |
| *Comphonema angustatum* |  |  |  |  |  |  |  | + |  |  | + | + | + |  |  |  |  |  | + | + |  |  |  |  |  |
| *Gomphonema parvulum* |  | + |  | + | + | + |  | + |  | + | + | + | + | + | + | + | + |  | + |  |  | + |  |  |  |
| *Gomphomema parvulum var.subellipticum* |  |  |  |  |  | + |  |  |  |  |  |  |  |  |  |  |  |  |  |  |  |  |  |  |  |
| *Gomphonema olivaceum* |  |  |  |  |  |  |  |  |  | + |  |  |  | + | + |  |  |  | + |  |  |  |  |  |  |
| *Gomphonema constiicum var.capitatum* |  | + |  |  | + | + | + | + |  | + | + | + | + | + | + | + | + | + | + | + | + | + |  |  | + |
| *Gomphonema abbreviatum* |  |  |  |  |  |  |  |  |  |  |  |  |  |  |  |  | + |  |  |  |  |  |  |  |  |
| *Gomphonema gracile* |  |  |  |  |  |  |  |  |  |  | + |  |  |  |  |  |  |  |  |  |  |  |  |  |  |
| *Gomphomema turris* |  |  |  |  | + |  |  | + | + | + |  | + |  |  |  | + | + | + |  |  |  |  |  |  | + |
| *Cophonema intricatum var. pumlium* |  |  |  |  |  | + |  |  |  |  |  |  |  | + |  |  |  |  |  |  |  |  |  |  |  |
| *Cophonema intricatum* |  |  |  |  |  |  |  |  |  |  | + |  |  |  |  |  |  | + |  |  |  |  |  |  |  |
| *Gomphomema* sp. | + |  | + | + | + |  |  |  | + | + | + |  | + |  |  |  |  |  |  |  | + |  |  |  |  |
| *Cyclotella stelligera* |  |  | + |  |  |  | + | + |  |  |  |  |  |  |  |  |  |  |  | + |  | + |  |  |  |
| *Cyclotella meneghiniana* |  |  |  | + |  |  |  |  |  | + |  | + | + |  |  |  | + |  |  | + |  | + |  |  |  |
| *Cyclotella* sp. | + | + | + | + | + | + | + | + | + | + | + | + | + | + |  |  |  |  |  | + | + | + | + |  |  |
| *Pinnularia nobilis* | + |  |  |  |  |  |  |  |  |  | + | + | + |  |  |  |  |  |  |  |  |  |  |  |  |
| *Pinnularia interrupta* | + |  |  |  | + | + | + | + |  |  | + | + | + | + | + |  | + | + | + | + |  |  |  |  |  |
| *Pinnularia gibba* |  |  |  |  |  |  |  |  |  |  | + |  |  | + | + |  |  |  |  |  |  |  |  |  |  |
| *Pinnularia molaris* |  |  |  |  |  |  |  |  |  |  |  | + |  |  | + |  |  |  |  |  |  |  |  |  |  |
| *Pinnularia microstauron* |  |  |  |  |  |  |  |  |  |  | + |  | + |  |  |  |  |  | + |  |  |  |  |  |  |
| *Pinnularia viridis* |  |  |  |  |  |  |  |  |  |  |  | + |  |  |  |  |  |  |  |  |  |  |  |  |  |
| *Pinnularia major* |  |  |  |  |  |  |  |  |  |  | + |  | + |  |  |  |  |  | + |  |  |  |  |  |  |
| *Pinnularia mesolepta* |  |  |  |  |  |  |  |  |  |  |  |  |  | + | + |  |  |  | + |  |  |  |  |  |  |
| *Pinnularia divergentissima* |  |  |  |  |  |  |  |  |  |  |  |  |  |  |  | + |  |  |  |  |  |  |  |  |  |
| *Pinnularia* sp. |  |  |  |  |  |  |  |  |  |  | + | + | + |  | + |  |  |  | + |  |  |  |  |  |  |
| *Diploneis elliptica* |  |  |  |  |  |  |  |  |  |  |  |  |  |  |  |  | + |  |  |  |  |  |  |  |  |
| *Caloneis ventricosa* |  |  |  |  |  |  |  |  |  |  | + | + |  |  |  |  |  | + |  |  |  |  |  |  |  |
| *Caloneis ventricosa var.truncatula* |  |  |  |  |  |  |  |  |  |  |  |  | + |  |  |  |  |  |  |  |  |  |  |  |  |
| *Caloneis schumanniana* |  |  |  |  |  |  |  |  |  |  | + |  | + |  |  |  |  | + |  |  |  |  |  |  |  |
| *Gyrosigma acuminatum* |  |  |  |  |  |  |  | + |  | + | + | + | + |  |  |  |  |  |  |  |  |  |  |  |  |
| *Cymbella lunata* |  |  |  |  |  |  | + | + |  |  |  |  | + |  |  |  | + | + | + |  |  |  |  |  |  |
| *Gyrosigma spencerii* |  |  |  |  |  | + |  | + |  |  |  |  | + | + | + | + | + | + | + | + |  |  |  |  |  |
| *Gyrosigma* sp. | + |  |  |  |  | + | + |  | + |  |  | + | + | + |  |  | + | + | + |  | + | + |  |  |  |
| *Eunotia pectinnalis* | + | + |  | + |  | + |  | + |  |  | + | + | + | + | + |  |  |  | + |  |  |  |  |  | + |
| *Eunotia pectinnalis* var.*minor* |  |  |  |  |  | + |  |  |  |  |  |  |  |  |  |  |  |  | + |  |  |  |  |  |  |
| *Eunotia sudetica* |  |  |  |  |  |  |  |  |  |  |  |  |  |  |  |  | + |  |  |  |  |  |  |  |  |
| *Eunotia* sp. | + |  |  | + | + | + |  | + | + | + | + | + | + | + |  | + | + |  | + | + |  |  |  |  |  |
| *Surirella robusta* | + |  |  | + |  |  | + | + | + |  | + | + | + | + | + | + | + | + | + | + |  | + |  | + | + |
| *Surirella caoronii* |  |  |  | + |  |  | + |  |  |  |  | + | + | + |  | + | + | + |  | + |  |  |  |  |  |
| *Surirella robusta var. splendida* | + |  |  |  |  |  |  |  |  |  |  |  |  | + | + |  |  |  |  |  |  |  |  |  |  |
| *Surirella ovata* |  |  |  |  |  |  |  |  |  |  |  |  |  |  |  |  |  | + |  |  |  |  |  |  |  |
| *Surirella ovata var.pinnata* |  |  |  |  |  |  |  | + |  |  |  |  |  | + |  |  |  |  |  |  |  |  |  |  |  |
| *Surirella linearis* |  |  |  |  |  |  |  | + |  |  |  | + | + | + | + |  |  |  | + |  |  |  |  |  | + |
| *Surirella spiralis* |  |  |  |  |  |  |  |  |  |  |  |  |  |  | + |  | + |  |  |  |  |  |  |  |  |
| *Surirella angustata* |  |  |  |  |  |  |  |  |  |  | + |  | + |  |  |  |  |  |  |  |  |  |  |  |  |
| *Surirella* sp. |  |  |  |  |  |  |  | + |  |  |  |  |  |  |  |  |  |  |  |  |  |  |  |  |  |
| *Nitzschia linearis* |  | + |  |  |  |  | + | + | + | + | + | + | + | + | + | + | + | + | + | + | + | + |  |  |  |
| *Nitzschia palea* | + | + |  | + | + | + |  | + | + | + | + | + | + | + | + | + | + | + | + | + | + | + |  |  | + |
| *Nitzschia amphibia* |  |  |  |  |  |  |  | + |  | + | + | + | + | + | + |  | + |  | + | + | + | + | + |  |  |
| *Nitzschia denticula* |  |  |  |  |  |  |  |  |  |  | + |  |  |  |  |  |  |  |  |  |  |  |  |  |  |
| *Fragilaria capucina* |  | + |  | + |  | + | + | + | + | + |  | + | + | + | + | + | + | + | + | + | + | + |  |  |  |
| *Fragilaria vaucheriae* |  |  |  |  |  |  |  |  |  |  |  |  |  |  |  |  |  |  |  |  |  | + |  |  |  |
| *Fragilaria intermedia* |  | + |  |  |  |  |  |  |  |  |  |  |  |  |  |  |  |  |  |  |  |  |  |  | + |
| *Fragilaria* sp. |  |  |  |  |  |  |  |  |  |  |  |  |  | + |  |  |  |  |  |  |  |  |  |  |  |
| *Navicula gracilis* |  |  |  |  |  |  |  |  |  |  |  |  |  |  | + |  |  |  |  |  |  |  |  |  |  |
| *Navicula placentula* |  |  |  |  |  |  |  |  |  |  |  |  |  |  |  |  |  |  |  |  |  |  |  |  | + |
| *Navicula cincta* |  |  |  | + | + |  |  | + | + | + | + | + | + | + | + |  | + |  | + | + |  |  |  |  |  |
| *Navicula minama* |  |  |  | + |  |  |  |  |  | + |  |  | + |  |  |  |  | + |  | + |  |  |  |  |  |
| *Navicula vanheurckii* |  |  |  |  |  |  |  |  | + | + |  |  |  |  |  | + |  |  |  |  |  |  |  |  |  |
| *Navicula bacillum* |  |  |  |  |  |  |  |  |  |  | + |  |  | + |  |  | + |  |  |  |  |  |  |  |  |
| *Navicula rhynchocephala* |  |  |  |  |  |  |  |  |  |  |  |  |  | + | + |  |  |  |  |  |  |  |  |  |  |
| *Navicula pupula* |  |  |  |  |  | + |  | + |  | + |  | + | + | + | + |  | + | + | + |  |  | + |  |  |  |
| *Navicula pupula var. cpitata* |  |  |  |  |  | + |  | + |  |  | + | + |  |  |  |  |  |  |  |  |  |  |  |  |  |
| *Navicula pupula var. rectangularia* |  |  |  |  |  |  |  |  |  |  | + | + |  | + |  | + | + |  | + |  |  |  |  |  |  |
| *Navicula protracta* |  |  |  |  |  |  |  |  |  |  |  |  |  |  |  |  |  |  |  |  |  |  |  | + | + |
| *Navicula dicephala* |  |  |  |  |  |  |  |  | + |  |  |  | + |  |  |  |  |  |  |  |  |  |  |  |  |
| *Navicula cuspidata* |  |  |  |  |  |  | + | + |  |  |  | + | + | + | + |  | + | + | + | + | + | + |  |  |  |
| *Navicula schoenfeldii* |  |  |  |  |  |  |  | + |  | + |  |  |  |  |  |  |  | + |  |  |  |  |  |  | + |
| *Navicula cari* |  |  |  |  |  |  |  |  |  |  |  |  |  |  | + |  |  |  | + |  |  |  |  |  |  |
| *Navicula anglica* |  |  |  |  |  |  |  |  |  |  | + |  |  | + | + |  | + |  | + |  |  |  |  |  |  |
| *Navicula simples* |  | + |  |  |  |  |  | + | + | + | + | + | + | + | + | + | + | + | + | + | + |  |  | + | + |
| *Navicula* sp. | + | + | + | + |  | + |  | + | + |  |  | + | + | + |  | + | + |  | + |  | + | + | + |  |  |
| *Navicula radiosa* |  |  |  |  |  |  |  |  |  |  |  |  | + | + | + |  | + |  |  |  |  |  |  |  |  |
| *Tabellaria fenestrata* |  |  |  |  |  |  |  |  |  |  |  |  | + |  |  |  |  |  |  |  |  |  |  |  |  |
| *Anomoeoneis sphaerophora* |  |  |  |  |  |  |  |  |  |  |  | + |  |  |  |  |  | + |  |  |  |  |  |  |  |
| *Cymatopleura elliptica* |  |  |  |  |  |  |  | + |  |  |  |  |  |  | + |  |  |  |  | + |  |  |  |  |  |
| *Cymatopleura solea* |  |  |  |  |  |  |  |  |  |  | + |  |  |  |  |  |  |  | + |  |  |  |  |  |  |
| *Cymatopleura* sp. |  |  |  | + | + |  |  | + | + | + |  |  | + | + | + |  |  |  | + |  |  |  |  |  |  |
| *Synedra amphicephala* |  |  |  |  |  |  |  |  |  |  |  | + |  | + |  |  |  |  |  |  |  |  |  |  |  |
| *Synedra acus* | + | + | + | + |  |  |  | + |  | + | + | + | + | + | + |  | + | + | + | + | + | + | + |  | + |
| *Synedra ulna var. constracta* |  |  |  |  |  |  |  |  |  |  |  |  |  |  |  |  |  |  |  |  |  |  |  | + |  |
| *Synedra ulna* |  | + |  | + | + | + |  | + | + | + | + | + | + | + | + | + | + | + | + | + | + | + | + |  | + |
| *Synedra* sp. | + | + |  | + |  |  | + | + | + | + | + | + | + | + |  | + | + |  |  | + | + |  |  |  |  |
| *Melosira granulata* | + | + | + | + |  | + | + | + | + |  | + | + | + | + | + | + | + | + | + | + | + | + | + |  | + |
| *Melosira granulata var. angustissima* | + | + | + | + |  | + | + | + |  | + | + | + | + | + | + | + | + | + | + | + | + | + | + |  | + |
| *Melosira granulata var. angustissima f.spiralis* | + |  |  |  | + |  |  |  |  |  |  | + | + |  |  |  |  |  |  |  |  | + |  |  |  |
| *Melosira varians* |  |  |  |  |  |  |  | + | + | + | + |  | + | + | + | + | + | + |  | + | + |  |  |  | + |
| *Microcystis incerta* |  |  |  |  |  | + |  |  |  |  |  |  |  |  |  |  | + | + |  |  |  |  |  |  |  |
| *Microcystis novacekii* | + | + |  |  | + | + | + |  |  |  |  |  |  |  |  |  |  |  |  |  |  |  |  |  |  |
| *Microcystis wesenbergii* | + | + | + | + | + | + |  | + |  |  |  |  |  |  |  |  |  |  |  |  |  | + |  |  |  |
| *Microcystis aeruginosa* | + | + | + | + | + | + | + |  |  |  |  | + |  |  |  |  |  |  |  | + |  |  |  |  |  |
| *Microcystis pseudofilamentosa* |  |  |  |  |  |  |  | + |  |  |  |  |  |  |  |  |  |  |  |  |  |  |  |  |  |
| *Microcystis flos-aquae* |  | + | + |  | + |  |  |  |  |  | + |  |  |  |  |  |  |  |  |  |  |  |  |  |  |
| *Microcystis viridis* | + | + | + | + | + |  | + |  |  |  |  |  |  |  |  |  |  |  |  |  |  |  |  |  |  |
| *Microcystis smithii* | + | + | + |  | + | + | + |  |  |  |  |  |  |  |  |  |  |  |  |  |  |  |  |  | + |
| *Microcystis marginata* |  |  |  |  |  | + |  |  |  |  |  |  |  |  |  |  |  |  |  | + |  |  |  |  | + |
| *Microcystis firma* | + |  |  |  |  | + | + |  |  |  |  |  |  |  |  |  |  |  |  |  |  |  |  |  | + |
| *Microcystis elabens* |  |  |  |  |  | + |  |  |  |  |  |  |  |  |  |  |  |  |  |  |  |  |  |  |  |
| *Microcystis pallida* |  |  |  |  |  |  |  |  |  |  |  |  |  |  |  |  |  |  |  |  |  | + |  |  |  |
| *Microcystis* sp. | + | + | + | + | + | + | + | + |  |  | + | + |  |  | + |  |  |  |  | + | + | + | + |  |  |
| *Oscillatoria orinces* | + |  | + | + |  | + | + | + |  |  |  | + | + | + |  | + | + |  |  |  | + | + |  |  |  |
| *Oscillatoria animalis* |  |  |  |  |  |  |  |  |  |  |  |  |  |  |  |  |  |  |  |  |  |  |  |  | + |
| *Oscillatoria anguina* |  |  |  |  |  |  |  |  |  |  |  |  |  |  | + |  |  |  |  |  |  |  |  |  |  |
| *Oseillatoriaii agardhii* |  |  | + | + | + |  | + | + |  |  |  |  | + |  |  |  |  |  |  |  |  |  |  |  |  |
| *Oscillatoria fraca* | + |  | + | + | + |  |  | + |  |  |  |  |  |  |  |  |  |  |  |  |  |  |  |  |  |
| *Oscillatoria tenuis* | + |  | + |  | + | + | + |  |  |  | + | + | + |  | + |  | + | + |  | + | + | + |  |  |  |
| *Oscillatoria simplicissima* |  |  |  |  |  |  |  |  |  |  |  |  |  |  |  |  |  |  |  |  |  |  |  |  | + |
| *Oscillatoria rubeccens* | + |  |  |  |  |  |  |  |  |  |  |  |  |  |  |  |  |  |  |  |  |  |  |  |  |
| *Oscillatoria* sp. | + | + | + | + | + | + | + | + | + | + | + | + | + | + | + |  | + | + | + |  | + |  |  |  |  |
| *Merismopedia elegans* |  |  |  |  |  |  |  |  |  |  |  |  |  |  |  |  |  | + |  |  |  |  | + |  |  |
| *Merismopedia minima* |  |  |  |  |  |  |  |  |  |  |  | + |  |  |  |  |  |  |  |  |  |  |  |  |  |
| *Merismopedia* sp. | + | + |  |  |  |  | + |  |  |  | + | + |  | + |  |  |  |  |  |  |  | + |  |  |  |
| *Phomidium tenue* |  |  |  |  |  |  | + |  |  |  |  |  |  |  |  |  |  |  |  |  |  | + |  |  |  |
| *Phormidiuan acutissimum* |  |  |  |  | + |  |  |  |  |  | + |  |  |  |  |  |  |  |  |  |  |  |  |  |  |
| *Phormidiuan* sp. |  |  |  |  |  |  |  |  |  | + |  |  |  |  |  |  | + |  | + | + |  | + |  |  |  |
| *Tychonema granulatum* |  |  |  |  |  |  |  |  |  |  |  |  |  |  |  |  |  |  |  |  | + | + |  |  |  |
| *Chroococcus limneticus* |  |  | + | + |  |  |  |  |  |  |  |  |  |  |  |  |  |  |  |  |  | + |  |  |  |
| *Chroococcus minutus* | + |  | + |  | + | + | + |  |  |  |  |  |  |  |  |  | + |  |  |  |  | + | + |  |  |
| *Chroococcus helveticus* |  |  | + |  |  |  |  |  |  |  |  |  |  |  |  |  |  |  |  |  |  |  |  |  |  |
| *Planktothrix prolifica* |  |  |  |  |  |  |  |  |  |  |  |  |  |  | + |  |  |  |  |  |  |  |  |  |  |
| *Planktothrix cryptovaginata* |  |  |  |  |  |  |  |  |  |  |  |  |  |  |  |  |  |  |  |  |  |  |  | + |  |
| *Planktothrix* sp. |  |  | + |  |  |  |  |  |  |  |  |  |  |  |  |  |  |  |  |  |  |  | + |  |  |
| *Spirulina major* |  |  |  |  |  |  |  |  |  |  |  | + |  |  |  |  |  |  |  |  |  |  |  |  |  |
| *Spirulina platensis* |  |  |  |  |  |  | + |  |  |  |  |  |  |  |  |  |  |  |  |  |  |  |  |  |  |
| *Arthrospira platensis* |  |  |  |  |  |  |  |  |  |  |  |  |  |  | + |  |  |  |  |  |  |  |  |  |  |
| *Arthrospira maxima* |  |  |  |  |  |  |  |  |  |  |  |  |  |  |  |  |  |  |  | + |  |  | + |  |  |
| *Limnothrix* sp. |  |  |  |  |  |  |  |  | + |  |  |  |  |  |  |  |  |  |  |  |  | + | + |  |  |
| *Cuspidothrix issatschenkoi* |  |  |  | + |  |  |  |  |  |  |  |  |  |  |  |  |  |  |  |  |  |  | + |  |  |
| *Dolichospermum* sp. |  |  | + | + |  |  |  |  |  |  |  |  |  |  |  |  |  |  |  |  |  |  |  |  |  |
| *Raphidiopsis sinensia* | + | + |  | + | + | + |  | + |  | + | + | + | + | + |  |  | + | + | + |  |  | + | + |  |  |
| *Raphidiopsis curvata* |  |  | + |  |  |  |  |  |  |  |  |  |  |  |  |  |  |  |  |  |  |  | + |  |  |
| *Coelosphaerium dubium* | + | + |  |  | + |  |  |  |  |  |  |  | + |  |  |  |  |  |  |  |  |  |  |  |  |
| *Aphanocapsa pulchra* |  |  |  |  |  |  | + |  |  |  |  |  |  |  |  |  |  |  |  |  |  |  |  |  |  |
| *Aphanocapsa elachista* | + |  |  |  |  | + | + |  |  |  |  |  |  |  |  |  |  |  |  |  |  |  |  |  |  |
| *Synechocystis willei* | + |  |  |  |  |  |  |  |  |  |  |  |  |  |  |  |  |  |  |  |  |  |  |  |  |
| *Annabaena cylindrica* |  |  | + |  |  |  |  |  |  |  |  |  |  |  |  |  |  |  |  |  |  |  | + |  | + |
| *Pseudanabaena galeata* |  |  |  |  |  |  |  |  |  |  |  |  |  |  |  |  |  |  |  |  |  |  | + |  |  |
| *Annabaena circinalis* | + |  | + |  |  |  |  |  |  |  |  |  | + |  |  |  |  | + |  |  |  |  | + |  | + |
| *Annabaena oscillariordes* |  |  | + |  |  |  |  |  |  |  |  |  |  |  |  |  |  |  |  |  |  |  |  |  |  |
| *Annabaena spiroides* |  |  |  | + |  |  | + |  |  |  |  |  |  |  |  |  |  |  |  |  |  |  |  |  | + |
| *Annabaena* sp. |  | + | + | + |  |  | + | + | + | + | + | + | + | + |  |  |  |  |  |  | + |  | + |  |  |
| *Pseudanabaena* sp. |  |  |  | + |  |  |  |  |  |  |  |  |  |  |  |  |  |  |  |  |  |  | + |  |  |
| *Nostoc minutum* |  |  |  |  |  | + |  |  |  |  |  |  |  |  |  |  | + |  |  |  |  |  |  |  |  |
| *Nostoc paludosum* |  |  |  |  | + |  |  |  |  |  |  |  |  |  |  |  |  |  |  |  |  |  |  |  | + |
| *Nostoc linckia* |  | + |  |  |  |  |  |  |  |  |  |  |  |  |  |  |  |  |  |  |  |  |  |  |  |
| *Nostoc* sp. |  | + |  |  |  | + |  |  |  |  |  |  |  |  |  |  |  |  |  |  |  |  |  |  |  |
| *Komvophoron anabaenoides* |  |  |  |  |  | + | + | + |  |  | + | + | + | + | + |  | + |  |  |  |  | + |  |  |  |
| *Woronichinia compacta* |  |  |  |  |  |  | + |  |  |  |  |  |  |  |  |  |  |  |  |  |  |  |  |  |  |
| *Phacus undulatus* |  |  |  |  |  |  |  |  |  |  |  |  |  |  |  |  | + | + | + | + |  |  |  |  |  |
| *Phacus hamatus* |  |  |  |  |  |  |  |  |  |  |  |  |  |  | + |  |  |  |  |  |  |  |  |  | + |
| *Phacus triqueter* |  |  |  |  |  |  |  |  |  |  |  |  |  |  |  |  |  |  | + |  |  |  |  |  |  |
| *Phacus acuminatus* |  |  |  |  |  |  |  |  |  |  | + |  |  |  |  |  |  |  |  |  |  |  |  |  |  |
| *Phacus longicauda* |  |  |  |  |  |  |  |  |  |  |  |  |  |  |  |  | + |  | + |  |  |  |  |  |  |
| *Phacus orbicularis* |  |  |  |  |  |  |  |  |  | + |  |  |  |  |  |  |  |  |  |  |  |  |  |  |  |
| *Phacus agilis* |  |  | + |  |  |  |  |  |  |  | + |  |  |  |  |  |  |  |  |  |  |  |  |  |  |
| *Phacus pyrum* |  |  |  |  |  |  |  |  | + |  |  |  |  |  |  |  |  |  |  |  |  |  |  |  |  |
| *Phacus lismorensis* |  |  |  |  |  |  |  |  |  |  |  |  |  |  |  |  | + |  |  |  |  |  |  |  |  |
| *Phacus* sp. |  |  |  |  |  |  |  |  |  |  |  | + |  |  |  |  |  |  |  |  |  |  |  |  |  |
| *Lepocinclis fusiformis* |  |  |  |  |  |  |  |  |  |  |  |  |  |  |  |  |  |  |  |  | + |  | + |  |  |
| *Lepocinclis steinii* |  |  |  |  |  |  |  |  |  |  |  |  |  |  |  |  |  |  | + | + |  |  |  |  |  |
| *Trachelomonas similis* |  |  |  |  |  |  |  | + |  |  |  |  |  |  |  |  | + |  |  |  |  |  | + |  |  |
| *Trachelomonas oblonga* |  |  |  |  |  |  |  |  |  |  |  | + |  |  |  |  |  |  | + |  |  |  | + |  |  |
| *Trachelomomas scabia* |  |  |  |  |  |  |  |  |  |  |  |  |  |  |  |  |  |  |  |  |  |  | + |  |  |
| *Trachelomonas volvocina* |  |  |  |  |  |  |  |  |  |  |  |  |  |  |  |  | + |  |  |  |  |  |  |  |  |
| *Trachelomonas granulosa* |  |  |  |  |  |  |  |  |  |  |  |  |  |  |  |  |  |  |  |  |  |  | + |  |  |
| *Trachelomonas adrupta* |  |  |  |  |  |  |  |  |  |  |  |  |  |  |  |  |  |  | + |  |  |  |  |  |  |
| *Trachelomonas klebsii* |  |  |  |  |  |  |  |  |  |  |  |  |  | + |  |  |  |  |  |  |  |  |  |  |  |
| *Trachelomonas* sp. |  |  |  |  |  | + |  | + |  |  | + | + | + |  |  |  |  |  |  |  | + | + | + |  |  |
| *Euglena prciformis* |  |  |  |  |  |  |  |  |  |  |  |  | + |  |  |  | + | + |  | + |  |  |  | + |  |
| *Euglena gracilis* |  |  |  | + | + |  |  |  |  |  | + |  |  |  |  |  |  |  |  |  |  | + | + |  |  |
| *Euglena oxyuris* |  |  |  |  |  |  |  | + |  |  |  | + | + | + |  |  | + |  |  |  | + | + |  |  | + |
| *Euglena caudata* |  |  |  |  |  |  |  |  |  |  |  |  |  |  |  |  | + | + | + |  |  |  |  |  |  |
| *Euglena polymrpha* |  |  |  |  |  |  |  |  |  |  |  | + |  |  |  |  | + | + |  | + | + | + | + |  |  |
| *Euglena spirogyra* |  |  |  |  |  |  |  | + |  |  |  |  |  |  |  |  |  |  | + |  |  |  |  |  |  |
| *Euglena gasterosteus* |  |  |  |  |  |  |  |  |  |  | + | + |  |  |  |  |  |  |  | + | + |  |  |  |  |
| *Euglena proxima* |  |  |  |  |  |  |  |  | + |  |  |  |  |  |  |  |  | + |  |  | + |  | + |  |  |
| *Euglena acus* |  |  |  |  |  |  | + | + |  |  |  | + | + |  |  |  | + | + | + |  | + |  |  |  | + |
| *Euglena viridis* |  |  |  |  |  |  |  |  |  |  |  |  |  |  |  |  |  |  |  |  | + |  |  |  |  |
| *Euglena* sp. |  |  |  |  |  | + |  | + | + |  | + | + | + |  |  | + | + |  | + | + |  | + | + |  |  |
| *Micractinium bornhemiensis* |  |  |  |  |  |  |  |  |  |  |  |  |  |  |  |  |  |  |  |  |  | + |  |  |  |
| *Pediastrum duplex* |  |  |  |  |  |  |  |  |  |  | + | + |  |  |  |  | + |  | + |  |  | + |  |  |  |
| *Pediastrum duplex var.gracillimum* |  |  |  |  |  |  |  |  |  |  |  |  |  |  |  |  |  |  |  |  |  | + |  |  |  |
| *Pediastrum simplex* |  |  |  |  | + |  |  |  |  |  |  | + | + |  |  |  |  |  | + |  | + | + | + |  |  |
| *Pediastrum simplex var.gracillimum* | + | + | + | + | + | + | + |  |  | + | + | + | + |  |  |  | + | + | + | + | + | + | + |  |  |
| *Pediastrum tetras* |  |  |  |  |  |  |  |  |  |  | + | + |  |  |  |  |  |  |  |  |  | + | + |  |  |
| *Pediastrum biradiatum* |  |  |  | + |  |  |  | + |  |  | + | + | + | + |  |  | + |  |  | + |  | + |  |  |  |
| *Pediastrum boryanum* |  |  |  |  |  |  |  |  |  |  |  | + | + |  |  |  |  |  |  |  |  |  |  |  |  |
| *Hyalotheca dissiliens* |  |  |  |  |  |  |  |  |  |  | + |  |  |  |  |  |  |  |  |  |  |  |  |  |  |
| *Desmidium swartzii* |  |  |  |  |  |  |  |  |  |  | + |  |  |  |  |  |  |  |  |  |  |  |  |  |  |
| *Xanthidium antilopaeum* |  |  |  |  |  |  |  |  |  |  | + |  |  |  |  |  |  |  |  |  |  |  |  |  |  |
| *Gonatozygon pilosum* |  |  |  |  |  |  |  |  |  |  | + |  |  |  |  |  |  |  |  |  |  |  |  |  |  |
| *Gonatozygon monotaenium* |  |  |  |  |  |  |  |  |  |  | + |  |  | + |  |  |  |  |  |  |  |  |  |  |  |
| *Actinastrum hantzschii* | + |  |  |  |  |  | + |  | + | + |  | + | + |  | + |  | + |  |  | + | + | + | + |  |  |
| *Dictyosphaerium pulchellum* |  | + | + |  | + | + | + |  |  |  |  |  |  |  |  |  |  |  |  | + |  | + | + |  |  |
| *Chodatella wratislaviensis* |  |  |  |  |  |  |  | + |  |  |  | + |  |  |  |  | + |  |  |  |  | + |  |  |  |
| *Coelastrum sphaericm* | + | + | + | + | + | + | + |  |  | + | + | + | + |  | + |  |  |  |  | + | + | + | + |  |  |
| *Coelastrum reticulatum* |  |  |  |  |  |  |  |  |  |  |  |  |  |  |  |  |  |  |  |  |  | + |  |  |  |
| *Coelastrum microporum* |  | + |  |  |  |  |  |  |  |  |  | + |  |  |  |  |  |  |  |  |  | + |  |  |  |
| *Coelastrum cambricum* |  |  |  | + |  |  |  |  |  |  | + | + |  |  | + |  |  |  |  |  |  | + |  |  |  |
| *Scenedesmus denticulatus* |  |  |  |  |  |  |  | + |  |  | + | + |  |  |  |  |  |  |  |  |  | + |  |  |  |
| *Scenedesmus dimorphus* |  |  |  |  |  |  |  |  |  |  | + | + | + |  | + |  |  | + |  | + |  | + | + |  |  |
| *Scenedesmus arcuatus* |  |  |  | + |  |  |  |  |  | + | + |  |  |  |  | + | + | + | + | + |  | + | + |  |  |
| *Scenedesmus carinatus* |  |  |  |  |  |  |  |  |  |  | + |  |  |  |  |  |  |  |  |  |  |  |  |  |  |
| *Scenedesmus perforatus* |  |  |  |  |  |  |  |  |  |  |  | + |  |  |  |  |  |  |  |  |  |  |  |  |  |
| *Scenedesmus bijuga* | + | + | + |  |  | + | + | + |  | + | + | + |  |  |  |  | + |  |  |  |  | + | + |  |  |
| *Scenedesmus quadricauda* | + | + |  |  |  | + |  | + |  | + | + | + |  |  | + |  | + | + | + | + | + | + | + |  |  |
| *Scenedesmus* sp. |  |  | + | + | + | + |  | + | + | + |  |  | + | + |  |  |  |  |  |  | + |  | + |  |  |
| *Tetrastrum hastiferum* |  |  |  |  |  |  |  |  |  |  |  | + |  |  |  |  |  |  |  |  |  | + |  |  |  |
| *Crucgenia apiculata* |  |  |  |  |  |  |  |  |  |  | + |  |  |  |  |  |  |  |  |  |  |  |  |  |  |
| *Crucgenia quadrata* |  |  |  |  |  |  |  |  |  |  | + |  |  |  |  |  |  |  |  |  |  |  |  |  |  |
| *Crucigenia lauterbornii* |  |  |  | + |  |  |  |  |  |  |  |  |  |  |  |  |  |  |  |  |  |  |  |  |  |
| *Crucgenia tetrapedia* |  |  |  |  |  |  |  | + |  |  | + |  |  |  |  |  |  |  |  |  |  |  |  |  |  |
| *Crucgenia apiculata* |  |  |  |  | + |  | + |  |  |  |  | + |  |  |  |  | + |  |  |  |  |  | + |  |  |
| *Stigeoclonium* sp. |  |  |  |  |  |  |  | + |  | + |  |  |  |  |  |  |  |  |  |  |  |  |  |  |  |
| *Ulothrix variabilis* |  |  |  |  |  |  |  |  |  |  | + |  |  |  |  |  |  |  |  |  |  |  |  |  |  |
| *Ulothrix zonata* |  |  |  | + |  |  | + | + |  | + | + | + | + | + | + |  | + | + | + | + |  |  |  |  |  |
| *Ulothrix* sp. |  |  |  |  |  |  |  |  | + | + |  |  |  |  |  |  |  |  |  |  |  |  |  |  |  |
| *Oedogonium* sp. |  |  |  |  | + |  |  | + |  |  | + | + |  | + |  |  |  |  |  |  |  |  |  |  |  |
| *Kirchneriella lunaris* |  |  |  |  |  |  |  |  |  |  | + |  |  |  |  |  |  |  |  |  |  |  |  |  |  |
| *Kirchneriella obesa* |  |  |  |  |  |  |  |  |  |  | + |  |  |  |  |  |  |  |  |  |  |  |  |  |  |
| *Spirogyra communis* |  | + |  |  |  |  |  |  |  |  |  | + | + | + |  |  | + |  |  | + |  |  |  |  |  |
| *Ankistrodesmus falcatus var. mirabilis* |  |  |  |  |  |  |  |  |  |  |  | + |  |  |  |  |  |  |  |  |  |  |  |  |  |
| *Chlorella vulgaris* | + | + | + | + | + | + | + |  | + |  | + | + | + |  | + |  | + |  | + | + | + | + | + |  |  |
| *Quadrigula chodatii* |  |  |  |  |  |  |  |  |  |  |  |  |  |  |  |  |  |  |  |  |  |  | + |  |  |
| *Characium limneticum* | + | + | + | + | + |  |  | + |  |  |  |  | + |  |  |  |  |  |  |  |  | + |  |  |  |
| *Tetraëdron trilobulatum* |  |  |  |  |  |  |  |  |  |  |  |  |  |  |  |  |  |  |  |  |  |  | + |  |  |
| *Tetraëdron minimum* |  |  |  |  |  |  |  |  |  |  |  |  |  |  |  |  |  |  |  |  |  | + | + |  |  |
| *Tetraëdron trigonum* |  | + |  |  |  |  |  |  |  |  |  |  |  |  |  |  | + |  |  |  |  |  |  |  |  |
| *Tetraedron regulare* |  |  |  |  | + |  |  |  |  |  |  | + |  |  |  |  |  |  |  |  |  | + | + |  |  |
| *Tetraedron caudatum* |  |  |  |  |  |  |  |  |  |  |  | + |  |  |  |  |  |  |  |  |  |  |  |  |  |
| *Schroederia nitzschioides* |  |  |  |  |  |  |  |  |  |  |  | + |  |  |  |  |  |  |  |  |  |  |  |  | + |
| *Schroederia setigera* |  |  |  |  |  |  |  |  |  |  |  |  |  |  |  |  |  |  |  |  |  |  | + |  |  |
| *Schroederia robusta* |  |  |  | + |  |  |  | + |  |  | + | + |  |  |  |  |  |  |  |  |  |  | + |  |  |
| *Oocystis borgei* |  |  | + |  |  |  |  |  |  |  | + |  |  |  |  |  |  |  |  |  |  |  |  |  |  |
| *Oocystis soltaria* | + |  |  | + | + |  | + |  |  |  | + |  |  |  |  |  |  |  |  |  |  | + |  |  |  |
| *Oocystis* sp. | + |  | + |  | + | + |  |  |  |  |  | + |  |  |  |  |  |  |  |  |  |  |  |  |  |
| *Eudorina elegans* | + | + | + | + | + | + | + | + |  |  | + |  | + |  |  |  | + | + |  | + |  | + |  |  | + |
| *Pandorina morum* | + |  | + | + | + |  | + |  |  |  |  | + | + |  |  |  |  |  |  | + | + | + | + |  |  |
| *Pleodorina californica* | + | + |  | + | + | + | + |  |  |  |  | + |  |  |  |  |  |  |  |  |  |  |  |  |  |
| *Planctonema lauterbornii* |  |  | + |  |  |  | + |  |  |  |  |  |  |  |  |  | + |  |  |  |  |  | + |  |  |
| *Staurodesmus dejectus* |  |  |  |  | + |  |  |  |  |  |  |  |  |  |  |  |  |  |  |  |  |  |  |  |  |
| *Staurodesmus dejectus var.apiculatus* |  |  |  |  | + |  |  |  |  |  |  |  |  |  |  |  |  |  |  |  |  |  |  |  |  |
| *Staurodesmus connatus* |  |  |  |  |  |  |  |  |  |  |  |  |  |  |  |  | + |  |  |  |  |  |  |  |  |
| *Actinotaenium globosum* |  |  |  |  |  |  |  |  |  |  |  |  | + |  |  |  |  |  |  |  |  |  |  |  |  |
| *Cosnarium meneghinii* |  |  |  |  |  | + |  |  |  |  |  |  |  |  |  |  |  |  |  |  |  |  |  |  |  |
| *Cosnarium laeve* | + |  |  |  | + |  |  | + |  | + |  | + | + |  |  |  |  |  | + |  |  |  | + |  |  |
| *Cosmarium globosum* |  |  |  |  | + |  |  |  |  |  |  |  |  |  |  |  |  |  |  |  |  |  |  |  |  |
| *Cosmarium javanicum* |  |  |  |  |  |  |  |  |  |  |  | + |  |  |  |  |  |  |  |  |  |  |  |  |  |
| *Cosmarium quadrum* |  |  |  |  |  |  |  |  |  |  |  | + |  |  |  |  |  |  |  |  |  |  |  |  |  |
| *Cosmarium formosulum* |  |  |  |  |  |  |  | + |  |  |  | + |  | + |  |  |  |  |  |  |  | + |  |  |  |
| *Cosmarium reniforme* |  |  |  |  |  |  |  |  |  |  | + |  |  |  |  |  |  |  |  |  |  |  |  |  |  |
| *Cosmarium obtusatum* |  |  |  |  |  |  |  |  |  |  |  |  |  | + |  |  |  |  |  |  |  |  |  |  |  |
| *Cosnarium* sp. |  |  |  |  |  | + | + |  |  |  |  |  |  |  |  |  |  |  |  |  |  |  |  |  |  |
| *Pleurotaenium trabecula* |  |  |  |  |  |  |  |  |  |  | + |  |  |  |  |  |  |  |  |  |  |  |  |  |  |
| *Euastrum denticulatum* |  |  |  |  |  |  |  |  |  |  |  |  |  |  |  |  |  |  |  |  |  |  | + |  |  |
| *Spondylosium moniliforme* |  |  |  |  | + |  |  |  |  |  |  |  |  |  |  |  |  |  |  |  |  |  |  |  |  |
| *Staurastrum pingue* |  |  | + |  | + | + |  |  |  |  |  |  |  |  |  |  |  |  |  |  |  |  |  |  |  |
| *Staurastrum gracile* |  |  | + | + | + |  |  |  |  |  | + |  |  |  | + |  |  |  |  |  |  | + |  | + |  |
| *Staurastrum gemelliparum* |  |  |  |  |  |  |  |  |  |  |  |  |  |  |  |  |  |  |  |  | + |  |  |  |  |
| *Staurastrum inflexum* |  |  |  |  |  |  |  |  |  |  |  |  |  |  |  |  |  |  |  |  |  | + |  |  |  |
| *Staurastrum manfeldtii* |  |  |  |  | + | + | + |  |  |  |  |  |  |  |  |  |  |  |  |  |  | + |  |  |  |
| *Chlamydomonas globosa* |  |  |  |  |  |  |  |  |  |  |  | + |  |  |  |  |  |  |  |  |  |  |  |  |  |
| *Chlamydomonas* sp. | + |  |  |  |  |  |  | + |  |  | + | + |  |  |  |  |  |  | + |  |  | + | + |  |  |
| *Mougeotias* sp. |  |  |  |  |  |  |  |  |  |  | + |  |  |  |  |  |  |  |  |  |  |  |  |  |  |
| *Klebsormidium* sp. |  |  | + | + |  |  |  |  |  | + |  | + |  |  |  |  | + |  | + |  | + | + |  |  |  |
| *Palmellococcus miniatus* | + | + |  | + |  | + |  |  |  |  | + |  |  |  |  | + | + | + | + |  |  |  |  |  |  |
| *Treubaria crassispina* |  |  |  |  |  |  |  |  |  |  |  |  |  |  |  |  |  |  |  |  |  |  |  | + |  |
| *Arthridesmus convergens* |  |  |  |  |  |  | + |  |  |  |  |  |  |  |  |  |  |  |  |  |  |  |  |  |  |
| *Volvox africanus* |  |  |  |  |  |  |  |  |  |  | + |  |  |  |  |  |  |  |  |  |  |  |  |  |  |
| *Microspora abbreviata* |  |  |  |  |  |  |  |  |  |  |  |  |  |  | + |  |  |  |  |  |  |  |  |  |  |
| *Microspora* sp. |  |  |  |  |  |  |  |  |  |  |  |  |  |  | + |  |  |  |  |  |  |  |  |  |  |
| *Raphidonema nivale* |  |  |  |  | + | + |  | + |  |  |  | + |  |  |  |  | + | + | + | + |  |  |  |  | + |
| *Closterium dianae* |  |  |  |  |  |  |  |  |  |  |  |  |  |  |  |  | + |  |  |  |  |  |  |  |  |
| *Closterium ehrenbergii* |  |  |  |  |  |  |  |  |  |  |  |  |  | + |  |  |  |  |  |  |  |  |  |  |  |
| *Closterium venus* |  |  |  |  |  |  |  |  |  |  |  |  |  |  | + |  |  |  |  |  |  |  |  |  |  |
| *Closterium acerosum* |  |  |  |  |  |  |  |  |  |  |  | + | + | + |  |  |  |  |  | + |  | + |  |  |  |
| *Closterium kuetzingii* |  |  |  |  |  |  |  |  |  |  |  |  |  |  |  |  |  |  |  |  |  |  |  | + |  |
| *Closterium nematodes* |  |  |  |  |  |  |  |  |  |  |  |  |  |  |  |  |  |  | + |  |  |  |  |  |  |
| *Closterium lineatum* |  |  |  |  |  |  |  | + |  |  |  |  |  |  |  |  |  |  | + |  |  |  |  |  |  |
| *Closterium leibleinii* |  |  |  |  |  |  |  |  |  |  |  |  |  |  |  |  | + |  |  |  |  |  |  |  |  |
| *Closterium parvulum* |  |  |  |  |  |  |  |  |  |  |  |  |  |  | + |  |  |  |  |  |  |  |  |  |  |
| *Closterium* sp. |  |  |  |  | + |  |  |  |  |  |  |  |  |  |  |  |  |  |  |  |  |  |  |  |  |
| *Closterium gracile* |  |  |  |  |  |  |  |  |  |  | + |  |  |  |  |  | + | + |  |  |  | + | + |  |  |
| *Glenodinium pulvisculus* |  |  |  |  |  |  |  |  |  |  |  | + |  |  |  |  |  |  |  | + |  |  |  |  |  |
| *Glenodinium gymnodinium* |  |  |  |  |  |  |  |  |  |  |  |  |  |  |  |  |  | + | + | + |  | + |  |  |  |
| *Peridinium pusillum* | + |  |  |  |  |  |  |  |  |  | + |  | + |  |  |  |  |  |  |  |  | + | + |  |  |
| *Peridinium bipes* |  |  | + |  |  |  |  |  |  |  | + |  |  |  |  |  |  |  |  |  |  | + | + |  |  |
| *Peridinium elpatiewskyi* |  |  |  |  |  |  |  |  |  |  | + |  | + |  |  |  |  |  |  |  | + | + | + |  |  |
| *Peridinium* sp. | + |  |  | + |  |  |  |  | + |  | + | + | + |  |  |  | + |  |  | + | + | + | + |  |  |
| *Ceratium hirundinella* |  | + | + | + | + |  |  | + |  |  |  |  |  |  |  | + | + |  |  |  |  |  |  |  | + |
| *Cryptomonas erosa* | + |  | + |  |  |  |  |  |  |  |  | + |  |  |  |  |  |  |  |  |  |  |  |  |  |
| Cryptomonas *acuta* | + | + | + | + | + | + | + |  |  |  |  |  | + |  |  |  |  |  |  |  | + | + | + |  |  |
| Cryptomonas *ovata* | + | + | + | + | + | + | + | + | + | + | + | + | + |  |  | + | + | + | + | + | + | + | + |  |  |
| *Dinobryon* bavaricum |  |  |  |  |  |  |  |  |  |  | + |  | + |  |  |  | + |  |  |  |  |  |  |  | + |
| *Mallomonas* sp. |  |  |  |  |  |  |  |  |  |  | + |  |  |  |  |  |  |  |  |  |  |  |  |  |  |
| *Mallomonas caudata* |  |  |  |  |  |  |  |  |  |  | + |  |  |  |  |  |  |  |  |  |  |  |  |  |  |
| *Synura* sp. |  |  | + |  |  |  |  | + |  |  |  | + |  |  |  |  |  |  |  |  | + |  |  |  |  |
