# Supplementary material for: Effect of the ten‐year fishing ban on change of phytoplankton community structure: Insights from the Gan River
Source: Ecol Evol. 2024 Aug 29;14(9):e70217. doi: 10.1002/ece3.70217 (PMC11362611; doi:10.1002/ece3.70217)
Supplement: Supplementary file 5 — Table S5. [file ECE3-14-e70217-s005.docx]

Table S5 Environmental factors in wet and dry periods in the middle and lower reaches of the Gan River. Sampling site codes are as in Table S1. WP: wet period

| Sampling sites | Sampling time | WT | DO | EC | TDS | Sal | pH | ORP | Chla | CODMn | NH3-N | TP | TN | Cu | SD |
| --- | --- | --- | --- | --- | --- | --- | --- | --- | --- | --- | --- | --- | --- | --- | --- |
| XN | WP | 34.612 | 13.333 | 478.167 | 0.260 | 0.190 | 8.937 | -1.033 | 3.897 | 3.2 | 0.574 | 0.050 |  | 0.00274 | 1.010 |
| YS | WP | 33.036 | 6.900 | 607.333 | 0.340 | 0.250 | 7.763 | 10.967 | 2.583 | 3 | 0.209 | 0.040 |  | 0.00273 |  |
| FY | WP | 31.929 | 6.767 | 607.000 | 0.350 | 0.253 | 7.587 | 2.500 | 1.283 | 2.900 | 0.270 | 0.100 |  | 0.001 | 0.800 |
| MY | WP | 24.403 | 8.300 | 80.767 | 0.050 | 0.037 | 7.647 | 35.400 | 0.640 | 2.300 | 0.200 | 0.034 |  | 0.001 |  |
| YZ | WP | 26.894 | 7.100 | 383.500 | 0.323 | 0.240 | 7.680 | 31.333 | 0.813 | 2.04 | 0.1738 | 0.041 |  | 0.000748 |  |
| WZ | WP | 27.655 | 7.300 | 319.267 | 0.200 | 0.143 | 7.610 | 21.600 | 1.817 | 3.5 | 0.107 | 0.106 |  | 0.00135 |  |
| YF | WP | 26.528 | 7.533 | 302.667 | 0.193 | 0.140 | 7.617 | 29.633 | 0.997 | 2.100 | 0.130 | 0.028 | 1.552 | 0.001 |  |
| SG | WP | 28.161 | 7.167 | 330.200 | 0.200 | 0.147 | 7.423 | 36.600 | 0.953 | 3.300 | 0.222 | 0.047 | 1.601 | 0.001 |  |
| GA | WP | 30.077 | 6.667 | 402.000 | 0.240 | 0.170 | 7.503 | 27.733 | 1.123 | 3.3 | 0.26 | 0.176 |  | 0.001 |  |
| SC | WP | 27.953 | 7.433 | 186.733 | 0.117 | 0.080 | 7.447 | 19.433 | 0.887 | 1.700 | 0.200 | 0.010 | 0.650 | 0.001 | 0.200 |
| JG | WP | 22.603 | 8.667 | 68.000 | 0.047 | 0.030 | 7.873 | 12.367 | 0.700 | 1.500 | 0.121 | 0.020 | 0.430 | 0.002 |  |
| YX | WP | 31.369 | 7.933 | 414.033 | 0.240 | 0.177 | 8.077 | -11.867 | 0.320 | 1.600 | 0.214 | 0.030 | 1.080 | 0.001 | 2.800 |
| JA | WP | 31.409 | 7.333 | 347.267 | 0.203 | 0.147 | 7.710 | -4.600 | 0.500 | 2.400 | 0.209 | 0.040 | 1.140 | 0.001 | 2.100 |
| AF | WP | 27.880 | 7.667 | 211.067 | 0.127 | 0.093 | 7.367 | 19.400 | 1.000 | 1.700 | 0.172 | 0.030 | 0.880 | 0.010 | 0.750 |
| YFC | WP | 33.464 | 9.167 | 264.267 | 0.150 | 0.103 | 8.087 | -3.633 | 2.493 | 1.900 | 0.327 | 0.050 | 1.250 | 0.002 |  |
| QY | WP | 33.481 | 5.733 | 279.300 | 0.153 | 0.110 | 6.987 | 31.367 | 1.800 | 2.5 | 0.19 | 0.065 |  | 0.001 | 1.500 |
| LX1 | WP | 22.718 | 9.933 | 124.467 | 0.087 | 0.063 | 8.027 | 37.067 | 1.087 | 2.300 | 0.620 | 0.110 | 3.300 | 0.001 |  |
| LX2 | WP | 20.083 | 8.497 | 71.200 | 0.050 | 0.035 | 7.497 | 82.300 | 0.870 | 2.700 | 0.180 | 0.030 | 1.300 | 0.000 |  |
| ZS | WP | 33.396 | 7.900 | 285.767 | 0.160 | 0.113 | 7.670 | 12.000 | 0.673 | 2.180 | 0.050 | 0.050 |  | 0.020 | 2.160 |
| FC | WP | 34.277 | 7.133 | 330.700 | 0.183 | 0.130 | 7.507 | -7.267 | 0.693 | 2.400 | 0.106 | 0.012 |  | 0.002 | 0.700 |
| TH | WP | 31.787 | 7.500 | 264.167 | 0.150 | 0.110 | 7.480 | -4.900 | 1.737 | 1.8 | 0.11 | 0.057 |  | 0.001 | 1.650 |
| WA | WP | 31.609 | 7.200 | 290.167 | 0.167 | 0.123 | 7.750 | -3.233 | 1.037 | 2.6 | 0.14 | 0.107 |  | 0.001 | 1.800 |
| XG | WP | 32.781 | 7.567 | 307.500 | 0.173 | 0.127 | 7.630 | -10.333 | 0.923 | 2.1 | 0.2 | 0.078 |  | -1 | 1.383 |
| JS | WP | 32.969 | 7.500 | 288.133 | 0.160 | 0.120 | 7.560 | 4.433 | 0.617 | 2.000 | 0.027 | 0.040 |  | 0.002 | 2.600 |
| XJ | WP | 32.781 | 7.033 | 288.833 | 0.160 | 0.120 | 7.457 | -9.267 | 0.613 | 2.3 | 0.274 | 0.05 | 1.70 | 0.003 | 3.250 |
| SC | DP | 21.373 | 10.367 | 194.700 | 0.137 | 0.100 | 7.523 | 51.233 | 0.527 | 1.500 | 0.152 | 0.010 | 0.790 | 0.019 | 0.250 |
| JG | DP | 17.738 | 9.733 | 69.533 | 0.053 | 0.037 | 7.643 | 32.967 | 0.453 | 1.900 | 0.109 | 0.020 | 0.500 | 0.001 |  |
| YX | DP | 21.266 | 9.000 | 496.067 | 0.343 | 0.257 | 8.277 | 16.533 | 0.243 | 2.600 | 0.085 | 0.050 | 1.030 | 0.005 | 3.000 |
| JA | DP | 20.047 | 9.700 | 425.667 | 0.307 | 0.223 | 8.467 | 15.433 | 0.170 | 3.400 | 0.268 | 0.090 | 1.170 | 0.027 | 2.950 |
| AF | DP | 20.258 | 8.133 | 412.833 | 0.297 | 0.217 | 8.097 | 7.167 | 1.073 | 3.900 | 0.177 | 0.070 | 0.890 | 0.003 |  |
| YFC | DP | 18.354 | 9.200 | 281.000 | 0.213 | 0.150 | 7.750 | 76.033 | 2.913 | 3.800 | 0.159 | 0.110 | 1.740 | 0.003 |  |
| QY | DP | 19.392 | 9.033 | 800.000 | 2.580 | 0.440 | 7.850 | 89.433 | 1.213 | 2.1 | 0.09 | 0.03 |  | 0.001 | 1.500 |
| MY | DP | 19.528 | 9.667 | 101.367 | 0.073 | 0.050 | 8.063 | 36.200 | 0.630 | 1.000 | 0.070 | 0.030 | 0.350 | 0.050 |  |
| YZ | DP | 20.937 | 10.667 | 601.000 | 0.423 | 0.320 | 8.140 | 35.133 | 0.283 | 2.160 | 0.286 | 0.028 |  | 0.001 |  |
| WZ | DP | 21.570 | 9.533 | 298.500 | 0.207 | 0.157 | 8.307 | 19.700 | 0.530 | 2.1 | 0.494 | 0.044 |  | 0.00094 |  |
| SG | DP | 22.865 | 8.667 | 657.333 | 0.443 | 0.337 | 7.657 | 36.267 | 0.383 | 2.900 | 0.270 | 0.031 | 1.335 | 0.002 |  |
| YF | DP | 22.992 | 8.667 | 721.667 | 0.487 | 0.367 | 7.637 | 46.767 | 0.573 | 2.900 | 0.270 | 0.031 | 1.335 | 0.002 |  |
| GA | DP | 21.385 | 8.800 | 690.000 | 0.480 | 0.360 | 8.133 | -8.400 | 5.920 | 2.4 | 0.06 | 0.077 |  | 0.00004 |  |
| XN | DP | 20.224 | 7.965 |  |  |  | 8.010 |  | 3.355 | 3.1 | 0.057 | 0.05 |  | 0.00242 | 0.950 |
| YS | DP | 19.223 | 7.100 |  |  |  | 7.225 |  | 3.960 | 3.4 | 0.025 | 0.040 |  | 0.00395 |  |
| FY | DP | 19.081 | 7.900 |  |  |  | 8.000 |  | 1.665 | 2.500 | 0.110 | 0.100 |  | 0.001 | 0.800 |
| XJ | DP | 23.553 | 8.437 | 316.733 | 0.212 | 0.153 | 7.747 | 60.833 | 0.343 | 2.0 | 0.487 | 0.05 | 1.37 | 0.00118 | 1.225 |
| XG | DP | 22.892 | 8.930 | 326.100 | 0.221 | 0.160 | 7.767 | 55.100 | 0.600 | 1.8 | 0.03 | 0.016 |  | -1 | 3.750 |
| JS | DP | 25.902 | 7.313 | 352.633 | 0.225 | 0.163 | 7.123 | 62.600 | 0.260 | 1.400 | 0.186 | 0.056 |  | 0.012 | 1.750 |
| ZS | DP | 22.037 | 9.667 | 334.300 | 0.227 | 0.170 | 8.163 | 30.800 | 1.070 | 2.400 | 0.050 | 0.050 |  | 0.020 | 2.850 |
| FC | DP | 22.818 | 9.250 | 367.950 | 0.245 | 0.185 | 8.055 | 62.150 | 1.075 | 2.100 | 0.034 | 0.054 |  | 0.001 | 1.500 |
| TH | DP | 20.259 | 8.433 | 399.467 | 0.287 | 0.213 | 7.573 | 71.700 | 0.510 | 1.4 | 0.08 | 0.018 |  | -1 | 1.050 |
| WA | DP | 22.388 | 7.967 | 428.300 | 0.293 | 0.217 | 7.767 | 19.133 | 0.857 | 2 | 0.05 | 0.035 |  | -1 | 0.810 |
