# Supplementary material for: Effect of the ten‐year fishing ban on change of phytoplankton community structure: Insights from the Gan River
Source: Ecol Evol. 2024 Aug 29;14(9):e70217. doi: 10.1002/ece3.70217 (PMC11362611; doi:10.1002/ece3.70217)
Supplement: Supplementary file 6 — Table S6. [file ECE3-14-e70217-s004.docx]

**Table S6** Density and biomass of phytoplankton in the wet and dry periods in the middle and lower reaches of the Gan River. Sampling site codes are as in Table S1. WP: wet period; DP: dry period; MR: main stem of the Gan River; BR: tributary of the Gan River

|  | Sampling sites | Wet period | | Dry period | |
| --- | --- | --- | --- | --- | --- |
|  |  | Density(×10^5^cells/L) | Biomass(mg/L) | Density(×10^5^cells/L) | Biomass(mg/L) |
| BR | XN | 39.0467 | 7.4059 | 18.5096 | 2.5417 |
|  | LX1 | 2.99 | 0.7053 | 1.58 | 0.3076 |
|  | YFC | 1.6133 | 0.3788 | 2.4217 | 0.5764 |
|  | YS | 1.57 | 0.6605 | 4.3233 | 1.4539 |
|  | MYS | 1.4667 | 0.6233 | 0.95 | 0.3356 |
|  | AF | 0.93 | 0.425 | 0.5833 | 0.3202 |
|  | JG | 0.905 | 0.4858 | 0.58 | 0.2621 |
|  | QY | 0.78 | 0.2896 | 0.5817 | 0.3256 |
|  | SC | 0.71 | 0.2906 | 0.4167 | 0.1247 |
|  | WZ | 0.6417 | 0.2419 | 0.49 | 0.1433 |
|  | LX2 | 0.48 | 0.1005 | 0.23 | 0.0458 |
|  | YF | 0.4667 | 0.3854 | 0.365 | 0.1226 |
|  | YZ | 0.4317 | 0.099 | 0.12 | 0.0373 |
|  | SG | 0.4283 | 0.1572 | 0.6583 | 0.4039 |
|  | GA | 0.3433 | 0.1524 | 2.4133 | 0.8374 |
|  | FY | 0.2433 | 0.09 | 2.8733 | 1.4757 |
|  | JA | 0.1583 | 0.0309 | 0.115 | 0.038 |
|  | YX | 0.1433 | 0.0583 | 0.1817 | 0.1178 |
| MR | XG | 0.1267 | 0.0138 | 0.6117 | 0.0798 |
|  | XJ | 0.11 | 0.043 | 0.2517 | 0.0801 |
|  | FC | 0.0766 | 0.0073 | 0.4217 | 0.0953 |
|  | WA | 0.29 | 0.3675 | 0.26 | 0.0715 |
|  | ZS | 0.2567 | 0.0421 | 0.9083 | 0.1291 |
|  | TH | 0.7183 | 0.0685 | 0.2 | 0.0183 |
|  | JS | 1.0433 | 0.1389 | 0.0317 | 0.005 |
